# Multiple imputation methods for bivariate outcomes in cluster randomised trials

**DOI:** 10.1002/sim.6935

**Published:** 2016-03-14

**Authors:** K. DiazOrdaz, M. G. Kenward, M. Gomes, R. Grieve

**Affiliations:** ^1^ Department of Medical StatisticsLondon School of Hygiene and Tropical MedicineKeppel StreetLondonW1C 7HTU.K.; ^2^ Department of Health Services Research and PolicyLondon School of Hygiene and Tropical Medicine15‐17 Tavistock PlaceLondonWC1H 9SHU.K.

**Keywords:** multiple imputation, cluster randomised trials, bivariate outcomes, missing data

## Abstract

Missing observations are common in cluster randomised trials. The problem is exacerbated when modelling bivariate outcomes jointly, as the proportion of complete cases is often considerably smaller than the proportion having either of the outcomes fully observed. Approaches taken to handling such missing data include the following: complete case analysis, single‐level multiple imputation that ignores the clustering, multiple imputation with a fixed effect for each cluster and multilevel multiple imputation.

We contrasted the alternative approaches to handling missing data in a cost‐effectiveness analysis that uses data from a cluster randomised trial to evaluate an exercise intervention for care home residents.

We then conducted a simulation study to assess the performance of these approaches on bivariate continuous outcomes, in terms of confidence interval coverage and empirical bias in the estimated treatment effects. Missing‐at‐random clustered data scenarios were simulated following a full‐factorial design.

Across all the missing data mechanisms considered, the multiple imputation methods provided estimators with negligible bias, while complete case analysis resulted in biased treatment effect estimates in scenarios where the randomised treatment arm was associated with missingness. Confidence interval coverage was generally in excess of nominal levels (up to 99.8%) following fixed‐effects multiple imputation and too low following single‐level multiple imputation. Multilevel multiple imputation led to coverage levels of approximately 95% throughout. © 2016 The Authors. Statistics in Medicine Published by John Wiley & Sons Ltd.

## 1 Introduction

In cluster randomised trials (CRTs), the unit of random allocation is a group of individuals (e.g. a school or a hospital) rather than the individual subjects. It is a common study design in the health and social sciences, especially for evaluations of interventions that operate at a group level, manipulate the socio‐physical environment or cannot be delivered at an individual level. It is well known that observations within each cluster are correlated 1, and that analyses that ignore this homogeneity within clusters can result in overestimation of the precision of the treatment effects, possibly leading to inappropriate inferences being drawn. Appropriate statistical techniques for CRTs are well developed and include mixed models and generalised estimating equations 2.

A common problem that compromises the validity of the results is that of missing data. The validity of inferences from incomplete data depends on the process that leads to data being missing, the so‐called missing data mechanism, also known as *missingness mechanism* or *missing data process* 3, section 3.2]. The missing data mechanism is characterised by the conditional distribution of the probability of missingness, given the data. A classification of the missing data mechanisms according to the assumed model for the probability of non‐response was introduced by Rubin 4. A process is said to be missing completely at random (MCAR) if the probability of non‐response is completely independent of any other variable, whether measured or not. A process is classified as missing at random (MAR) if the probability of non‐response is conditionally independent of the unobserved data given the observed data. Processes that are neither MCAR nor MAR are called missing not at random (MNAR).

For missing data mechanisms that satisfy MAR, valid inferences can be obtained using likelihood‐based or Bayesian analyses of the complete cases 3, Part III]. However, moment‐based estimators, such as those that use generalised estimating equations are, without special modification, only valid with more stringent conditions about the missing data mechanism, namely, that the missingness is independent of the outcome given the covariates in the model 5.

With partially observed clustered data, by far, the most common approach is to only analyse the complete cases (CCA) 6. However, when two or more outcomes are analysed jointly, the proportion of complete cases is often smaller than the complete cases corresponding to each outcome in turn. This is an important issue as multivariate outcomes are common in clinical trials. Examples include clinical trials of psychological interventions and those in cardiology, which often focus on non‐fatal cardiovascular events, in addition to time‐to‐event. Our paper uses cost‐effectiveness analyses (CEA) that use data from CRTs as an illustrative example. Most CEA that use individual‐level data from clinical trials have observations with incomplete information 7.

A common approach for obtaining valid inferences with incomplete data under the MAR assumption is to undertake multiple imputation (MI) 4. In some circumstances, essentially when the analysis and imputation models coincide, MI principally replicates a likelihood analysis. Nevertheless, an advantage of MI is that unlike conventional likelihood analyses, it can incorporate so‐called auxiliary variables that are not included in the analysis model but which are related to both the missing values and to the probability of observations being missing. Incorporating such auxiliary variables makes the underlying MAR assumption more plausible.

From a theoretical perspective, it is known that for CRTs, the imputation method should accommodate the multilevel structure of the data. A failure to do so may lead to invalid inferences 8. Unfortunately, multilevel MI (MMI) is not yet available as a standard implementation in commonly used statistical packages. Hence, analyses using MI in the CRT settings commonly avoid such imputation strategies and use instead single‐level imputation (SMI) methods 6. A systematic review of CEA that use CRT data 9 found that only 5% of studies included used MI, of which none accounted for the clustering.

An alternative approach that has been previously recommended in the literature is including the cluster as a fixed effect in the imputation model (FMI) 10, 11. This has the advantage of being easily implemented in widely available MI software.

The aim of this paper is to investigate and compare the performance of these different MI strategies for handling missing bivariate outcome data in CRTs over a wide range of missingness mechanisms that are dependent on individual and cluster‐level variables. We do this by first applying the methods to a cost‐effectiveness study that used data from a published CRT (Section 3). Then, a simulation study with a full‐factorial design is presented in Section 4. We close with a few points of interpretation and discussion in Section 5.

## 2 Multiple imputation

Multiple imputation breaks down the analysis of incomplete data into a number of steps. We first need to distinguish between two statistical models. The first is the analysis model that would have been used had the data been complete. This is called the *substantive model* or *model of interest*. The second model, called the *imputation model*, is used to describe the conditional distribution of the missing data given the observed. For hierarchical data, this conditional distribution must reflect the multilevel nature of the data.

The MI algorithm proceeds by fitting the imputation model to the observed data and taking Bayesian draws from the posterior distribution of its model parameters. Missing data are then imputed from the imputation model, using the parameters previously drawn. These steps are repeated a fixed *M* number of times, to obtain *M* *completed* data sets. The substantive model is then fitted to the multiple data sets separately, producing *M* sets of parameter and covariance estimates, which are combined using Rubin's formulae 4 to produce a single MI estimate of the substantive model parameters and associated covariance matrix. Under the MAR assumption, this will produce consistent estimators and, in the absence of auxiliary variables, is asymptotically (as *M* increases) equivalent to maximum likelihood 12, 13.

Sampling from the approximate predictive distribution of the missing data as described earlier can be performed in several ways. Two broad approaches can be identified; the first approach jointly models incomplete variables, by sampling from an underlying joint predictive distribution 13, 14. In the second approach, referred to as full‐conditional specification (FCS) or *chained equations*, draws from the joint distribution are approximated using a sampler consisting of a set of univariate models for each incomplete variable conditional on all the other variables 15. In the motivating example and simulations presented here, both approaches are used for ease of implementation. For SMI and fixed cluster effects models, which are also essentially single‐level, the FCS method is used. The FCS approach is not well‐suited to proper multilevel MI and so, for these imputations, a joint modelling algorithm assuming multivariate normality is used 13. In our settings, because both outcomes are continuous, and modelled with normal linear regressions, the FCS algorithm is equivalent to a Gibbs sampler that draws from a multivariate normal distribution, and hence equivalent to a joint MI algorithm 16, 17, 18.

Having outlined the generic MI procedure, we now set out the details of the relevant imputation models to be compared here. Let *Y*_1,*ijk*_ and *Y*_2,*ijk*_ be the two continuous outcomes with missing data, corresponding to the *i*‐th individual in cluster *j* of a two‐arm cluster trial. Assume that *J* clusters are allocated to each treatment *k*∈{0,1}, and that there are *n*_*j*_ individuals in each cluster *j*, for *j* = 1,…,*J*. Let *k* indicate treatment allocation, *k* = 1, if the cluster is allocated to intervention, and 0 otherwise.

Let *X*_*ijk*_ denote the vector of fully observed variables, individual and cluster level, to be included in the imputation model. This includes the variables in our model of interest and any other auxiliary variables, and may be different in each treatment arm. The imputation models compared here are regression models of the outcomes on the covariates in the substantive model and the auxiliary variables, fitted separately within each treatment arm, to allow for different covariance structure.

The single‐level imputation model (used in SMI) can be written as

$$\begin{array}{c} Y_{1,\mathrm{ijk}}=\beta_{1,0k}+X_{\mathrm{ijk}}\beta_{1,X}+e_{1,\mathrm{ijk}}\\ Y_{2,\mathrm{ijk}}=\beta_{2,0k}+X_{\mathrm{ijk}}\beta_{2,X}+e_{2,\mathrm{ijk}} \end{array}\;\;\;\;\;\;\;\;\begin{array}{c} \left(\;\;\begin{array}{c} e_{1,\mathrm{ijk}}\\ e_{2,\mathrm{ijk}} \end{array}\;\;\right)∼N\left[\;\left(\;\begin{array}{c} 0\\ 0 \end{array}\;\right),\left(\begin{array}{cc} \sigma_{1k}^{2} & \rho_{k}\sigma_{1k}\sigma_{2k}\\ \rho_{k}\sigma_{2k}\sigma_{1k} & \sigma_{2k} \end{array}\right)\right] \end{array}$$

With SMI, the imputed values are drawn from the conditional distribution of the missing observations given the observed data, ignoring any dependency between observations within a cluster not explained by the cluster‐level auxiliary variables included in the model. Therefore, the single‐level imputation model does not properly represent the conditional distribution of the missing data given the observed data.

The effect of clustering can be incorporated either as a fixed or random effect. Firstly, we include a cluster fixed effect in the imputation model (corresponding to FMI):

$$\begin{array}{cc} Y_{1,\mathrm{ijk}} & =\beta_{1,0k}+X_{\mathrm{ijk}}\beta_{1,X}+\beta_{1,\mathrm{jk}}+e_{1,\mathrm{ijk}}\\ Y_{2,\mathrm{ijk}} & =\beta_{2,0k}+X_{\mathrm{ijk}}\beta_{2,X}+\beta_{2,\mathrm{jk}}+e_{2,\mathrm{ijk}} \end{array}$$

where *β*_*ℓ*,*jk*_ are the fixed cluster‐effect coefficients, different from 0 only if the observation *i* belongs to cluster *j* in treatment group *k*, for *j* = 1,…,*J*. To avoid over‐parameterisation, *β*_*ℓ*,1*k*_=0, for *k*∈{0,1},*ℓ*∈{1,2}, making the first cluster in each treatment arm the reference category. The error terms (*e*_1,*ijk*_,*e*_2,*ijk*_) are assumed to be bivariate normal as before. Missing outcomes will be imputed from the conditional normal distribution given the other outcome, if observed, and the covariates and auxiliary variables, which must all be at the individual level, with a mean determined by the fixed effect for that cluster.

We note that this parameterisation of the fixed‐effects imputation model may result in biased estimates when there is a high proportion of clusters with completely missing outcomes. This is because FMI imputes empty clusters from the distribution of the reference cluster, as the fixed effect for the empty cluster cannot be estimated. When this is the case, the imputer must choose the reference cluster carefully. In particular, we should choose the cluster that has a cluster‐mean closest to the randomised‐group mean.

An alternative to the FMI is to include a cluster random effect in the imputation model (corresponding to MMI):

(1) $$\begin{array}{c} Y_{1,\mathrm{ijk}}=\beta_{1,0k}+X_{\mathrm{ijk}}\beta_{1,X}+u_{1,\mathrm{jk}}+e_{1,\mathrm{ijk}}\\ Y_{2,\mathrm{ijk}}=\beta_{2,0k}+X_{\mathrm{ijk}}\beta_{2,X}+u_{2,\mathrm{jk}}+e_{2,\mathrm{ijk}} \end{array}\;\;\;\;\;\;\;\;\begin{array}{c} \left(\;\;\begin{array}{c} u_{1,\mathrm{jk}}\\ u_{2,\mathrm{jk}} \end{array}\;\;\right)∼N\left[\;\left(\;\begin{array}{c} 0\\ 0 \end{array}\;\right),\left(\;\;\begin{array}{cc} \tau_{1k}^{2} & \phi_{k}\tau_{1k}\tau_{2k}\\ \phi_{k}\tau_{1k}\tau_{2k} & \tau_{2k}^{2} \end{array}\;\;\right)\;\right] \end{array},$$

again separately in each treatment group *k*. The individual‐level residuals (*e*_1,*ijk*_,*e*_2,*ijk*_) are assumed normally distributed and correlated as before, independently of (*u*_1,*jk*_,*u*_2,*jk*_), the cluster random effects.

Finally, complete case analysis (CCA) is also included in our simulations and example for comparative purposes.

### 2.1 Substantive model

In this paper, we assume that the substantive model is a bivariate linear random‐effects model where the only explanatory variable is treatment. This means that in what follows, the vector *X*_*ijk*_ of explanatory variables in the imputation models specified in the previous section contains only auxiliary variables. If, however, the substantive model includes baseline covariates, these must be included in the imputation model, as covariates if they are fully observed, or as dependent variables, if they themselves have missing values.

The substantive model is fitted to the data from both arms simultaneously, assuming common variance across the treatment arms. Let the cluster random effects be represented by the latent variables *u*_1,*jk*_ and *u*_2,*jk*_. The model can be written as follows:

(2) $$\begin{array}{cc} Y_{1,\mathrm{ijk}} & =\beta_{1,0}+\beta_{1}k+u_{1,\mathrm{jk}}+e_{1,\mathrm{ijk}}\\ Y_{1,\mathrm{ijk}} & =\beta_{2,0}+\beta_{2}k+u_{2,\mathrm{jk}}+e_{1,\mathrm{ijk}} \end{array}$$

where *β*_1_ and *β*_2_ represent the treatment effect on the corresponding outcome. The error term (*e*_1,*ijk*_,*e*_2,*ijk*_) and the cluster effects are assumed to be normally distributed:

$$\left(\;\;\begin{array}{c} e_{1,\mathrm{ijk}}\\ e_{2,\mathrm{ijk}} \end{array}\;\;\right)∼N\left[\;\left(\;\begin{array}{c} 0\\ 0 \end{array}\;\right),\left(\;\;\begin{array}{cc} \sigma_{1}^{2} & \rho \sigma_{1}\sigma_{2}\\ \rho \sigma_{1}\sigma_{2} & \sigma_{2}^{2} \end{array}\;\;\right)\;\right]\text{and}\left(\;\;\begin{array}{c} u_{1,\mathrm{jk}}\\ u_{2,\mathrm{jk}} \end{array}\;\;\right)∼N\left[\;\left(\;\begin{array}{c} 0\\ 0 \end{array}\;\right),\left(\;\;\begin{array}{cc} \tau_{1}^{2} & \phi \tau_{1}\tau_{2}\\ \phi \tau_{1}\tau_{2} & \tau_{2}^{2} \end{array}\;\;\right)\;\right]$$

where *σ*_1_,*σ*_2_ are the individual‐level standard errors, *ρ* is the individual‐level correlation between *Y*_1_ and *Y*_2_ and *τ*_1_,*τ*_2_ and *ϕ* are the standard errors and correlation of the two cluster random effects, respectively.

## 3 Motivating example: the OPERA study

We illustrate our methods using the OPERA study (exercise for treating depression in care home residents). It was a CRT to evaluate the impact of a ‘whole home’ exercise intervention on depressive symptoms in care home residents in England, aged 65years or over who are free of severe cognitive impairment 19. Clusters were randomly allocated to provide either a depression awareness training session for care home staff (control) or an exercise intervention delivered by a visiting physiotherapist (treatment). The intervention comprised twice weekly physiotherapist‐led exercise groups.

For the purpose of illustration, we look at the cost‐effectiveness data, which consisted of 798 individuals in 72 nursing homes. There were 31 clusters in the intervention and 41 in the control arm. As is common, the OPERA CRT had an imbalanced design; the number of participants per cluster varied from 5 to 20.

This paper considers costs (in Great British pounds, £) and health‐related quality of life completed via proxy (based on European Quality of Life questionnaire – EQ5D) recorded at three‐monthly intervals, for a period of 12months. These EQ5D data were used to obtain quality‐adjusted life years (QALYs) over 12months. Intra‐cluster correlation coefficients (ICCs) were high for QALYs (0.23 in the intervention and 0.08 in the control) but moderate for costs (0.03 for intervention and 0.10 in the control arm). While QALYs were approximately normally distributed, costs were positively skewed. The correlation between the outcomes was −0.11 in the control arm and −0.07 in the intervention.

The data set also includes the clinical primary outcome, depression, measured using the Geriatric Depression Score‐15 (GDS‐15), and baseline covariates at both cluster level –location and size of the home– and at the individual level –age, sex, ethnicity, being on antidepressants, years spent in formal education, cognitive impairment (Mini‐Mental State Examination Ű MMSE), physical function (Short Physical Performance BatteryŰSPPB), fear of falling, pain, social engagement, baseline GDS‐15 and baseline EQ5D (self‐completed and proxy).

We had 449 individuals with complete cost‐effectiveness data, 190 individuals with only missing QALYs at 12months, a further 110 with only costs missing and an additional 49 individuals with both outcomes missing. Table 1 reports the percentage of observations with missing cost‐effectiveness outcomes and baseline covariates, by treatment group. The number of clusters with one outcome completely missing was moderate: one intervention and six control clusters had QALYs completely missing (less than 10% of the total). There were no clusters with completely missing costs.

**Table 1:** Description of missing data in the OPERA study, by treatment group (top panel), and results from CEA by MI method: Incremental cost (£) and QALYs and INB (£) at 12months.

	Control group (Total *n*=446)		Intervention group (Total *n*=352)	
Outcome variables	Missing *n*	*%*	Missing *n*	*%*
Cost	82	18.4	77	21.9
QALY	159	35.7	80^*a*^	22.7
CEA by MI method				
Outcome	CCA	SMI^*b*^	FMI^*c*^	MMI^*b*^
Incremental cost	256.4(442.0)	166.67(454.09)	27.4(548.8)	177.27(440.88)
Incremental QALY	−0.04(0.04)	−0.05(0.03)	−0.02(0.05)	−0.04(0.05)
INB	−1148.9(920.5)	−1237.0(840.23)	−453.4(1137.2)	−978.5(1163.6)

One observation was removed from the data set before performing any analysis or MI, due to having missing age at baseline. This corresponded to an individual with missing QALY in the treatment arm.

Imputation models included age at baseline, sex and cluster size as auxiliary variables.

Imputation models included age at baseline and sex as auxiliary variables.

INB calculated at willingness to pay £20000.CCA, complete case analysis; SMI, single‐level imputation; FMI, fixed effect in the imputation model; MMI, multilevel multiple imputation; QALY, quality‐adjusted life year; CEA, cost‐effectiveness analysis; INB, incremental net monetary benefit.

The CEA assumes a model with linear additive treatment effects for both costs and QALYs, with no additional covariates. The corresponding effect of treatment, incremental QALYs *δ*_*Q*_ and incremental costs *δ*_*C*_, is estimated from a bivariate normal mixed model (2) 20. Cost‐effectiveness is then reported as the estimated incremental net monetary benefit (INB)

(3) $$\text{INB}(\lambda )=\lambda \delta_{Q}-\delta_{C}$$

where *λ* represents the decision‐makers' *willingness to pay* for a one unit gain in health outcome. Thus, the new treatment is cost‐effective if INB > 0. In the original study, the reported INB was calculated using *λ*= £ 20000, which is within the range of the cost‐effectiveness threshold recommended by the UK National Institute for Health and Care Excellence 21. As the INB is a linear combination of ${\hat{\delta }}_{C}$ and ${\hat{\delta }}_{Q}$, its variance can be calculated from the corresponding estimated variances and covariances, in the usual way.

### 3.1 Multiple imputation methods for the OPERA study

We now apply the alternative MI methods to the OPERA data set. For the purpose of illustration, we delete from the data set the single observation with missing age at baseline and consider age, sex and cluster size as completely observed baseline variables and use them as auxiliary variables in the imputations, as they are associated with the missingness and the outcomes. All MI strategies use the same baseline covariates as auxiliary variables in the imputation model, with one exception. Cluster size is dropped from the FMI approach, as cluster‐level variables cannot be used as explanatory variables in models using fixed cluster effects. Although costs are somewhat skewed, we do not log‐transform or perform post‐imputation rounding, as this has been shown to bias the associations 22, 23. Both outcomes, costs and QALYs, are included in all imputation models. The number of imputations in this example is 50.

We calculate INB on each multiply imputed data set using bivariate linear mixed models (2) and combine these results using Rubin's rules to obtain MI estimates. We construct normal‐based confidence intervals (CIs) around the MI estimate.

Single‐level imputation and FMI are implemented in R package mice, which uses the FCS algorithm. The number of iterations or cycles of the chained equations algorithm used is 50, as this appears to lead to satisfactory convergence for this data set. For the MMI, we use R package pan, with 1000 burn‐in iterations and imputed every 3000 to reduce auto‐correlation and improve convergence, as it is known that with large number of clusters and small ICCs, the Gibbs sampler is slowly mixing 8.

All three MI methods result in approximately 35 negative imputed costs per imputed set. Table 1 shows that the estimates of incremental QALYs, which has relatively high ICCs, are relatively insensitive to the choice of MI approach. By contrast, the incremental cost point estimate obtained by FMI is very different from the others. The standard errors across the two outcomes are different for each missing data approach but are relatively large compared with the size of the estimate. SMI produces smaller standard errors than those obtained with MMI and CCA. This is because costs and QALYs have a relatively large ICC in the OPERA data set, and we are looking at a between‐cluster estimator. As a consequence, there is an increased risk of type I error 24.

The choice of MI method, which mostly affects the way the variance of the missing data is modelled, affects the estimated SE. Nevertheless, for the OPERA study, all MI approaches lead to the same conclusion, that the OPERA intervention is not cost‐effective compared with the control treatment.

## 4 Simulation study

We now use a full‐factorial simulation study, to compare the performance of the MI methods across a wide range of circumstances typically found in CRTs. The simulation steps proceeded as follows: data generation, application of a missing data mechanism and estimation and inference for the treatment effect from the analysis after handling (or ignoring) the missing data. Finally, the behaviour of the treatment effect estimator is examined according to our chosen performance measures.

### 4.1 Data generation

We begin by selecting those factors anticipated to have an impact on the performance of the approaches for handling missing data, based on previous literature 24, 25: number of clusters per treatment arm and number of individuals per cluster (three settings); ICCs of the outcomes (four levels); and proportion of missing data (two levels) and missing data mechanism (four settings). The total number of clusters is 2*J*, with *n*_*j*_ individuals in cluster *j*, for *j*∈{1,…,*J*}, in each trial arm *k*∈{0,1}. The number and size of clusters are allowed to vary while maintaining the same expected sample size (*S* = 500). This sample size is typical of the sample sizes seen in CRTs, as a recent systematic review of CRTs published in medical journals reported the inter‐quartile range (IQR) of number of participants per arm as being [143–866] 26. Three different types of two‐arm CRT design are considered: (i) large number of clusters (*J* = 25) and few individuals per cluster (*n*_*j*_=10); (ii) small number of clusters (*J* = 5) and large cluster size (*n*_*j*_=50); and (iii) moderate number of clusters (*J* = 15) and variable number of individuals per cluster. The small and large number of clusters were also chosen to be close to the lower and upper quartiles of number of clusters reported by Ivers 26, which found an IQR of [12–52]. Following previous simulation studies 20, 27, the variable cluster size *n*_*j*_ is obtained by rounding a Gamma‐distributed random variable. This Gamma random variable has mean 20 and coefficient of variation $\mathrm{cv}=\frac{\text{SD(n)}}{\text{E(n)}}=0.5$. The full description of the simulation factors and their levels are summarised in Table 2. There are 3 × 4×2 × 2×4 = 192 simulated scenarios in total.

**Table 2:** Simulation design factors and chosen levels.

Factor		Levels		Values
ICC_1_ and ICC_2_		Low		(0.01, 0.01)	
		Moderate		(0.20, 0.05)	
		High		(0.20, 0.20)	
		Differential by outcome		(0.60, 0.01)	
Cluster design		Many small clusters		*J* = 25, *n*_*j*_=10	
		Few large clusters		*J* = 5, *n*_*j*_=50	
		Unbalanced		*J* = 15, variable size	
Missingness		Individual covariate		logit*π*_*ℓ*,*ij*_=*α*_0_+*η*_*X*_ *X*_*i*_	
mechanism		Cluster covariate		logit*π*_*ℓ*,*ij*_=*α*_0_+*η*_*W*_ *W*_*j*_	
		Both		logit*π*_*ℓ*,*ij*_=*α*_0_+*η*_*X*_ *X*_*i*_+*η*_*W*_ *W*_*j*_	
		Differential by treatment		logit*π*_*ℓ*,*ijk*_=*α*_0*k*_+*η*_*X*,*k*_ *X*_*ij*_+*η*_*W*,*k*_ *W*_*j*_	
Association between covariates		Low		*η*_*X*_=*η*_*W*_=*η* = 1	
and missingness		High		*η*_*X*_=*η*_*W*_=*η* = 2	
Probability of non‐response		Equal		20*%*	
		Different by outcome		30*%* for *Y*_1,*ij*_; 10*%* for *Y*_2,*ij*_	
Levels for missingness mechanisms that are differential by treatment arm					
		Association	Probability of non‐response		
		with		Different by outcome	
Level of association	Arm	missingness	Equal	For *Y*_1_	For *Y*_2_
Low	Control	*η*_*X*,0_=*η*_*W*,0_=1	20%	30%	10%
	Intervention	*η*_*X*,1_=*η*_*W*,1_=2	*35%*	*45%*	*20%*
High	Control	*η*_*X*,0_=*η*_*W*,0_=1.5	10%	15%	10%
	Intervention	*η*_*X*,1_=*η*_*W*,1_=3	*30 %*	*35%*	*30%*

^Note:^The top part of the table reports values for scenarios with missingness mechanisms, which do not differ by treatment arm; those corresponding to missingness mechanism which are differential by treatment arm, are reported at the bottom.

The numbers in italics are not simulation parameters but the approximate empirical rates of non‐response obtained after setting *α*_0_.

ICC, intra‐cluster correlation coefficient.

In each simulated scenario, a cluster‐level indicator is then created allocating half of the clusters to treatment and half to control. Then, for each subject *i* in cluster *j*, i.i.d standard normal individual‐level covariate *X*_*i*_ and cluster‐level variable *W*_*j*_ are generated. These are independent of treatment allocation *k* and are therefore thought of as pre‐randomisation variables. Then, bivariate normal outcome data (*Y*_1,*ijk*_,*Y*_2,*ijk*_) are generated separately by treatment arm as follows:

(4) $$Y_{1,\mathrm{ijk}}=100+120k+\beta_{w,1}W_{\mathrm{jk}}+\beta_{x,1}X_{i}+u_{1,\mathrm{jk}}+e_{1,\mathrm{ijk}}$$

(5) $$Y_{2,\mathrm{ijk}}=50+10k+\beta_{w,2}W_{\mathrm{jk}}+\beta_{x,2}X_{i}+u_{2,\mathrm{jk}}+e_{2,\mathrm{ijk}}$$

with (*e*_1,*ijk*_,*e*_2,*ijk*_)^⊤^∼N(**0**,*Σ*) and (*u*_1,*jk*_,*u*_2,*jk*_)^⊤^∼N(**0**,Φ), where $\Sigma =\left(\;\;\begin{array}{ll} \sigma_{1}^{2} & \rho \sigma_{1}\sigma_{2}\\ \rho \sigma_{1}\sigma_{2} & \sigma_{2}^{2} \end{array}\;\;\right)$is the level‐1 variance‐covariance matrix, with *σ*_1_=40, *σ*_2_=20 and *ρ* = 0.1 assumed constant across all scenarios. The level‐2 variance‐covariance matrix $\Phi =\left(\;\;\begin{array}{ll} \tau_{1}^{2} & \phi \tau_{1}\tau_{2}\\ \phi \tau_{1}\tau_{2} & \tau_{2}^{2} \end{array}\;\;\right)$is chosen so that the level of clustering, quantified by the ICC, takes different values, as specified in each scenario. The values for these ICCs, namely, 0.01, 0.05, 0.20 and 0.60 were based on approximation to the sample median 0.048 and IQR of ICCs, [0.016–0.124] with a maximum of 0.667, reported in a previous review of ICCs in medical research 28.

We note that while the variables *X* and *W* are associated with the outcomes, they are not included in the substantive model, and as such, they are auxiliary variables in the imputation models to follow.

The R function used to generate these data, by changing the levels of each factor accordingly, can be found in the Supporting Information File 2.

### 4.2 Missing data mechanisms

To generate the missing data for each outcome under the MAR assumption, we used four different missing data mechanisms, where the probability of missingness, denoted by *π*_*ℓ*,*ijk*_, with *ℓ*∈{1,2}, is such that the non‐response indicator *R*_*ℓ*,*ijk*_∼Bern(*π*_*ℓ*,*ijk*_), depends on *X*_*i*_ and/or *W*_*j*_, as displayed in Table 2. The coefficient *η* represents the strength of association between the covariates and missingness indicator *R*_*ℓ*,*ijk*_. We adjust *α*_0_ empirically to achieve the required expected probability of missing.

For both outcomes, individual‐level and cluster‐level covariates have the same level of association, *η*, with the missingness indicator, and thus we drop the subscripts *ℓ* and *X*,*W*. However, for the last of our missingness mechanisms, we allow *η* to differ between treatment arms. This represents a situation where there is an interaction between treatment and the covariate driving the missingness, that is, the treatment modifies strength of association between the covariate and non‐response, which may arise in clinical trials, because, for example, of side effects or lack of perceived efficacy in the intervention arm or disillusionment amongst those assigned to the control arm. We allow two settings; these are presented at the bottom of Table 2, together with the probabilities of non‐response, which also differ across treatment arms.

Non‐response rates are chosen to be moderate, to avoid situations where a high proportion of clusters have one or both outcomes completely missing.

For each simulated data set, non‐response indicators *R*_*ℓ*,*ijk*_ for each outcome *ℓ*∈{1,2} are independently drawn from a Bernoulli distribution with probabilities *π*_*ℓ*,*ijk*_ as specified in Table 2. Missing values are then generated to create the *observed* data set.

### 4.3 Implementation

For a simulation study of this size, it is important to balance computational time with efficiency of the methods. The number of imputations *M* is set to 10 29, although in practice, a higher number of imputed sets is recommended 10.

The MI methods using FCS, that is, SMI and FMI, are implemented using the mice package in R. The chained equations procedure was repeated for 10 cycles to produce a single imputed data set, following recommendations in 30. For the multilevel MI, Schafer's pan package in R is used 31. Details on the MCMC procedure used in pan can be found in 13. Briefly, a Gibbs sampler is used to simulate draws from the posterior distribution of the parameters, starting with the level 2 variances. For our simulations, we use non‐informative priors for regression parameters, diffuse inverse‐Wishart priors for variance components and impute on every 1000th iteration, after a 1000‐iteration burn‐in. In practice, one needs to monitor the convergence behaviour of the MCMC algorithm and modify the number of iterations between imputations and the burn‐in period accordingly.

After imputation, for which the two covariates are used as auxiliary variables, the substantive model, Equation (2), is applied to each multiply imputed data set to estimate treatment effect on *Y*_1_ and *Y*_2_ simultaneously. The estimates obtained using the analysis model in each of the *M* multiply imputed sets are then combined using Rubin's rules. CIs around the MI estimates are constructed using a normal distribution, instead of a *t*‐distribution with the small‐sample MI degrees of freedom 32. This is for comparability with the CIs obtained after a CCA, which are also constructed routinely using a normal approximation.

For each scenario, the whole simulation procedure (data generation, imposing missing values, imputation, analysing each of the imputed data sets using the substantive model and combining the resulting treatment effect estimates using Rubin's rules) is performed on each of the *N* = 1000 data sets to capture the behaviour in repeated samples.

### 4.4 Performance criteria

Let *β*_*ℓ*_ denote the true treatment effect on *Y*_*ℓ*_, with *ℓ*∈{1,2}, and ${\hat{\beta }}_{\ell ,\iota }$ the estimate obtained in the *ι* = 1,…,*N* replicated data set. The following criteria were used to measure the performance of the different MI strategies.

1. Confidence interval coverage rate: The percentage of times that the true parameter value is covered in the 95% CI.
2. Empirical bias, $B=\frac{1}{N}\overset{N}{\underset{\iota =1}{\sum }}\left({\hat{\beta }}_{\ell ,\iota }-\beta_{\ell }\right)$
3. Root‐mean‐square error (RMSE) $\sqrt{\frac{1}{N}\overset{N}{\underset{\iota =1}{\sum }}{\left({\hat{\beta }}_{\ell ,\iota }-\beta_{\ell }\right)}^{2}}$
4. Average width of confidence interval (AW): The distance between the average lower and upper CI limits across *N* CIs.

The performance of a procedure is regarded as poor if its coverage drops below 90% 33. When the procedure results in over‐coverage, there is an increased type II error probability. When coverage is close to 100%, extra caution should be taken when using that procedure 34, especially when coupled with wide CIs. Coverage close to the nominal value, along with narrow CIs, translates into greater accuracy and higher power.

### 4.5 Simulation results

**Figure 1:**
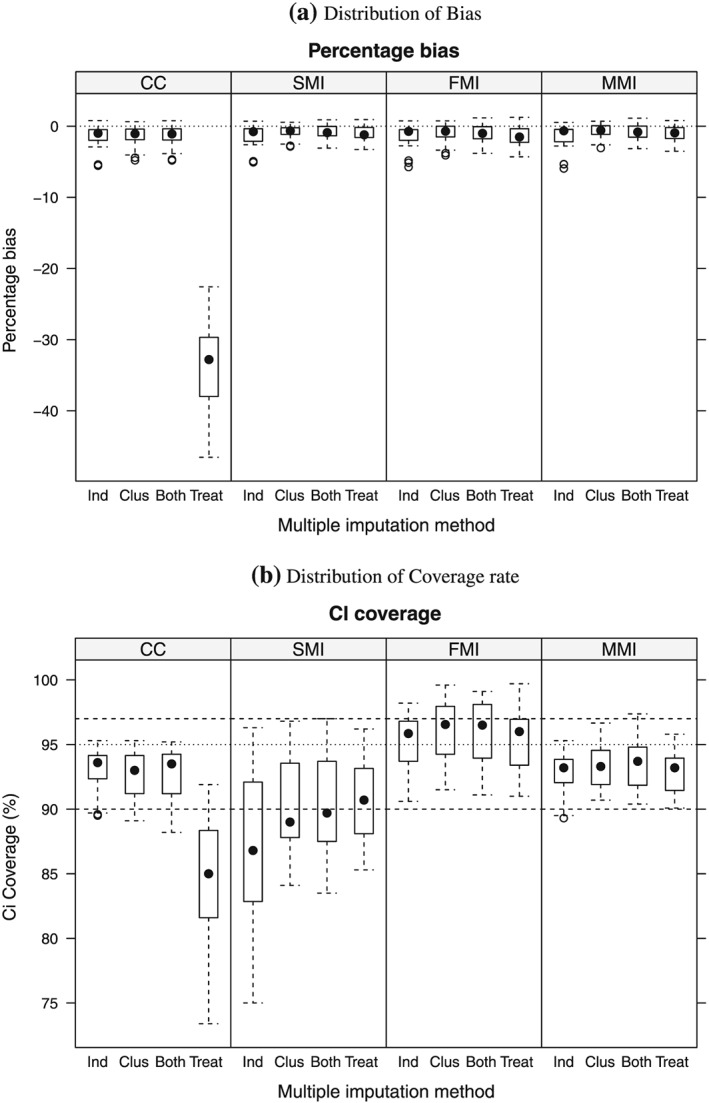
Boxplot of the distribution of (a) percentage bias and (b) coverage rate for treatment effect estimates on *Y*_1_, by analysis strategy (CCA, SMI, FMI and MMI), stratified by missingness mechanism, denoted by the columns Ind, individual covariate; Clus, cluster‐level covariate; Both and Treat, indicating the variables associated with missingness. Each box‐and‐whiskers plot represents 48 scenarios. The dotted black lines represent (a) no bias and (b) the nominal coverage rate, while the dashed lines represent minimum (90%) and maximum (97%) acceptable coverage rates. CCA, complete case analysis; SMI, single‐level imputation; FMI, fixed effect in the imputation model; MMI, multilevel MI; MI, multiple imputation.

**Figure 2:**
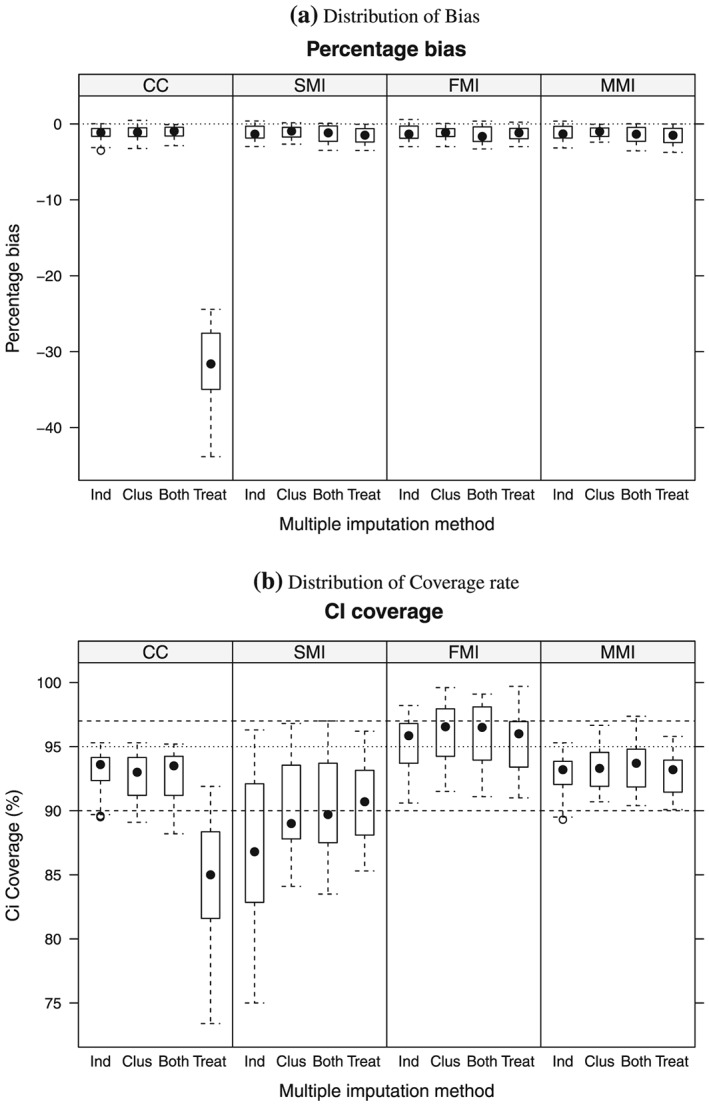
Boxplot of the distribution of (a) percentage bias and (b) coverage rate for treatment effect estimates on *Y*_2_, by analysis strategy (CCA, SMI, FMI, MMI), stratified by missingness mechanism, denoted by the columns Ind, individual covariate; Clus, cluster‐level covariate; Both and Treat, indicating the variables associated with missingness. Each box‐and‐whiskers plot represents 48 scenarios. The dotted black lines represent (a) no bias and (b) the nominal coverage rate, while the dashed lines represent minimum (90%) and maximum (97%) acceptable coverage rates. CCA, complete case analysis; SMI, single‐level imputation; FMI, fixed effect in the imputation model; MMI, multilevel multiple imputation.

Figures 1 and 2 present, respectively, the bias and coverage distribution for each method. Each box‐and‐whiskers plot shown represents 48 scenarios, stratified by missing data mechanism. The performance of the methods across the scenarios was similar for both *Y*_1_ and *Y*_2_.

For the first three missing data mechanisms shown (Table 2), where the missing data mechanism was not dependent on treatment arm, all approaches resulted in unbiased estimates across most of the scenarios. This is in line with theoretical results, as the variables associated with missingness are not associated with the treatment effect. However, for the scenario where the missingness mechanism is differential by treatment arm, the CCA produced substantially biased estimates across the scenarios considered. This corresponds to the situation where there is a different association between the covariate and the response indicator in each treatment arm, and this treatment‐covariate interaction is not accounted for when we condition on the complete cases. By contrast, the corresponding results for the MI estimates show negligible bias: in general, less than 3.5% and mostly within Monte Carlo error limits. To see this in more detail, see Table 3.

**Table 3:** Percentage bias for the estimated treatment effect on *Y*_1_ for scenarios corresponding to missingness mechanism is differential by treatment.

Design	*η*	Missingness	ICC	CCA	SMI	FMI	MMI
*J* = 25, *n*_*j*_=10	Low	.20,.20	0.01, 0.01	−24.8	−1.4	‐0.8	−0.8
			0.20, 0.05	−32.9	−1.5	−1.3	−1.0
			0.20, 0.20	−33.1	−1.6	−1.3	−1.0
			0.60, 0.01	−38.7	−1.4	−2.1	−1.4
		.30,.10	0.01, 0.01	−23.2	−1.3	−0.7	‐0.2
			0.20, 0.05	−31.0	−1.6	−1.7	−0.3
			0.20, 0.20	−31.1	−1.6	−1.7	−0.4
			0.60, 0.01	−35.9	−1.8	−3.5	−0.5
	High	.20,.20	0.01, 0.01	−28.2	−1.7	−2.3	‐1.7
			0.20, 0.05	−37.5	−1.7	−3.0	−2.0
			0.20, 0.20	−37.9	−1.9	−3.0	−1.9
			0.60, 0.01	−43.4	−1.5	−4.2	−2.6
		.30,.10	0.01, 0.01	−29.2	−1.3	−1.1	‐1.5
			0.20, 0.05	−39.3	−1.5	−2.3	−1.7
			0.20, 0.20	−39.7	−1.6	−2.3	−1.7
			0.60, 0.01	−46.5	−1.4	−4.3	−2.1
*J* = 5, *n*_*j*_=50	Low	.20,.20	0.01, 0.01	−25.2	0.1	−0.2	‐0.9
			0.20, 0.05	−31.1	−1.0	−1.5	−1.8
			0.20, 0.20	−31.5	−1.0	−1.5	−1.7
			0.60, 0.01	−32.7	−2.8	−3.7	−3.5
		.30,.10	0.01, 0.01	−24.5	−0.1	−0.4	−0.9
			0.20, 0.05	−30.2	−1.3	−1.7	−1.9
			0.20, 0.20	−30.7	−1.3	−1.7	−1.9
			0.60, 0.01	−31.7	−3.3	−4.0	−3.5
	High	.20,.20	0.01, 0.01	−29.2	0.0	−0.4	−0.4
			0.20, 0.05	−36.5	−1.1	−1.7	−1.3
			0.20, 0.20	−37.0	−1.2	−1.7	−1.2
			0.60, 0.01	−38.5	−2.9	−4.0	−3.2
		.30,.10	0.01, 0.01	−31.1	0.3	−1.5	−0.1
			0.20, 0.05	−38.9	−0.7	−1.7	−0.9
			0.20, 0.20	−39.5	−0.8	−1.6	−0.7
			0.60, 0.01	−41.0	−2.3	−4.3	−2.2
*J* = 15, unbalanced	Low	.20,.20	0.01, 0.01	−23.1	0.9	1.2	0.4
			0.20, 0.05	−30.3	0.4	0.6	0.0
			0.20, 0.20	−30.6	0.3	0.5	−0.1
			0.60, 0.01	−33.7	−0.6	−0.8	−1.1
		.30,.10	0.01, 0.01	−22.6	0.5	1.2	0.4
			0.20, 0.05	−29.6	−0.4	0.2	−0.2
			0.20, 0.20	−29.8	−0.5	0.3	−0.3
			0.60, 0.01	−33.0	‐2.0	−1.5	−1.2
	High	.20,.20	0.01, 0.01	−26.8	0.8	0.4	0.5
			0.20, 0.05	−35.4	0.3	−0.3	0.0
			0.20, 0.20	−35.8	0.2	−0.3	−0.1
			0.60, 0.01	−39.6	−0.5	−1.8	−1.1
		.30,.10	0.01, 0.01	−28.3	0.7	0.5	0.8
			0.20, 0.05	−37.6	−0.2	−0.6	0.3
			0.20, 0.20	−38.1	−0.4	−0.7	0.1
			0.60, 0.01	−42.6	−1.8	−2.7	−0.8

CCA, complete case analysis; SMI, single‐level imputation; FMI, fixed effect in the imputation model; MMI, multilevel MI; MI, multiple imputation; ICC,inter‐cluster correlation coefficient.

However, the alternative MI strategies result in very different variance estimates and consequently varying coverage rates. This is evident in the plots of CI coverage Figures 1 (b) and 2 (b). In general, SMI resulted in coverage lower than the nominal. This is particularly critical for scenarios with high ICCs (0.20 and above). The number and size of clusters also appear to be factors associated with low coverage rate. See Tables A4, A5 and A6 in the Supporting Information File 1. In contrast, fixed‐effects MI results in over‐conservative coverage for a range of scenarios, especially those where the ICCs are small and the number of clusters is large and the cluster‐level variable is associated with the missingness mechanism. In addition, wider CIs are obtained using FMI compared with those obtained using MMI, even when coverage was similar. See Tables A5, A6, A16 and A17 in the Supporting Information File 1. MMI results in acceptable coverage rates in most scenarios, but for scenarios where the number of clusters is relatively low (*J* = 5 per arm) and the clustering is high (≥0.2), coverage rates are only just above 90%. This is because the convergence of the Gibbs sampler depends on the degree to which the cluster random effects in the imputation model, Equation (1), can be estimated from the observed data 8. Convergence can be improved by increasing the burn‐in period for the sampler in the MMI software. For example, we re‐ran the scenario with *J* = 5 and the ICC=0.2 for both outcomes with differential missingness by treatment arms with *η* low and missing proportions 0.2 in each outcome. By increasing the burn‐in to 5000, CI coverage rate increased to 92.0%, compared with 90.9% reported in Table 4 for the same scenario. We therefore recommend that, when faced with small numbers of clusters, the burn‐in period is increased.

**Table 4:** Coverage rate (CR) and average width (AW) corresponding to confidence interval of the treatment effect estimate, when missingness is differential by treatment arm.

				CCA		SMI		FMI		MMI	
Design	*η*	Missingness	ICC	CR	AW	CR	AW	CR	AW	CR	AW
*J* = 25,	Low	.20,.20	0.01, 0.01	**8**1.2	18.6	95.5	17.9	*9*8.8	22.3	94.9	17.5
*n*_*j*_=10			0.20, 0.05	**8**4.7	27.6	92.6	26.2	96.5	31.2	93.1	27.4
			0.20, 0.20	**83.9**	27.7	92.8	26.3	96.3	31.2	93.1	27.2
			0.60, 0.01	91.3	56.0	90.7	51.4	95.1	58.7	94.4	56.9
		.30,.10	0.01, 0.01	**8**2.2	18.7	95.5	19.4	*99.7*	26.1	94.9	18.9
			0.20, 0.05	**85.2**	27.7	92.5	26.9	*9*7.5	34.0	93.0	28.2
			0.20, 0.20	**8**4.6	27.7	92.6	27.0	*9*7.5	34.0	92.4	27.9
			0.60, 0.01	91.7	56.0	90.7	50.8	95.2	60.1	93.5	57.5
	High	.20,.20	0.01, 0.01	**75.1**	17.4	95.6	17.3	*9*8.7	21.4	95.6	17.2
			0.20, 0.05	**8**1.2	26.7	91.8	26.2	96.8	30.6	93.8	27.4
			0.20, 0.20	**8**1.2	26.6	91.9	26.2	96.8	30.6	93.7	27.2
			0.60, 0.01	**8**9.7	55.2	91.0	52.3	94.6	58.4	94.4	56.9
		.30,.10	0.01, 0.01	**7**3.7	17.8	96.2	18.5	*9*9.1	23.4	95.8	18.1
			0.20, 0.05	**7**8.4	26.8	92.2	26.8	96.8	32.0	93.2	28.0
			0.20, 0.20	**7**8.6	26.8	92.3	26.8	96.8	32.1	93.6	27.8
			0.60, 0.01	**89.3**	55.3	90.4	52.2	95.0	59.1	94.4	57.4
*J* = 5,	Low	.20,.20	0.01, 0.01	**8**2.0	20.3	94.8	20.2	96.5	22.6	95.6	20.2
*n*_*j*_=50			0.20, 0.05	**8**7.4	48.1	**8**7.4	47.0	92.4	53.1	91.0	51.1
			0.20, 0.20	**8**7.2	48.4	**8**7.8	47.0	92.6	53.1	90.9	51.1
			0.60, 0.01	**8**9.9	116.1	**8**7.5	107.3	91.1	121.5	90.9	120.2
		.30,.10	0.01, 0.01	**8**3.2	20.3	94.8	21.8	*97.2*	25.4	95.3	21.7
			0.20, 0.05	**8**6.6	47.9	**8**7.2	46.3	92.1	54.3	90.3	51.2
			0.20, 0.20	**8**7.7	48.2	**8**7.2	46.4	92.4	54.1	90.2	51.0
			0.60, 0.01	**89.0**	115.7	**8**5.3	103.7	91.0	121.5	90.7	120.0
	High	.20,.20	0.01, 0.01	**7**5.6	19.4	94.8	19.8	96.1	21.9	94.1	20.0
			0.20, 0.05	**8**5.4	47.3	**8**8.9	47.9	92.0	52.7	91.4	51.3
			0.20, 0.20	**85.3**	47.5	**8**8.9	47.9	92.1	52.6	91.4	51.3
			0.60, 0.01	**89.7**	115.0	**8**7.9	110.2	91.2	121.0	91.0	120.2
		.30,.10	0.01, 0.01	**7**4.3	20.0	94.6	20.9	*97.1*	27.0	95.1	21.0
			0.20, 0.05	**8**5.1	47.6	**87.6**	47.7	92.8	54.3	90.1	51.7
			0.20, 0.20	**8**4.9	47.8	**8**7.5	47.7	92.4	53.8	90.2	51.6
			0.60, 0.01	**8**9.1	115.0	**8**6.9	108.4	91.0	123.7	90.8	120.9
*J* = 15,	Low	.20,.20	0.01, 0.01	**8**1.0	17.7	93.9	17.0	*9*7.4	21.0	93.2	16.9
unbalanced			0.20, 0.05	**85.9**	32.1	**89.9**	30.3	95.7	36.1	93.0	32.9
			0.20, 0.20	**85.9**	32.1	**89.7**	30.3	96.0	35.9	93.1	32.7
			0.60, 0.01	91.2	70.3	**8**9.6	63.7	94.3	74.0	93.5	72.4
		.30,.10	0.01, 0.01	**8**2.6	17.8	93.8	18.4	*98.5*	24.6	93.9	18.3
			0.20, 0.05	**8**5.7	32.1	**8**8.0	30.4	96.4	38.3	92.2	33.4
			0.20, 0.20	**85.5**	32.1	**8**8.2	30.4	96.2	38.1	91.5	33.1
			0.60, 0.01	91.9	70.5	**8**7.4	62.1	94.3	75.1	94.0	72.9
	High	.20,.20	0.01, 0.01	**7**4.9	16.6	93.5	16.7	*9*7.6	20.4	93.6	16.8
			0.20, 0.05	**83.0**	31.1	90.9	30.8	96.0	35.6	92.9	33.0
			0.20, 0.20	**83.0**	31.2	90.6	30.7	95.9	35.5	92.8	32.8
			0.60, 0.01	91.0	69.5	**89.7**	65.5	94.6	73.6	93.8	72.4
		.30,.10	0.01, 0.01	**7**3.4	17.1	94.3	17.5	*9*7.4	22.9	93.8	17.7
			0.20, 0.05	**8**2.5	31.4	90.7	30.8	96.0	37.3	93.5	33.4
			0.20, 0.20	**8**2.1	31.4	91.0	30.8	96.2	36.9	93.0	33.1
			0.60, 0.01	90.5	69.7	**8**9.7	64.5	94.0	74.4	93.8	72.8

*Note:* Bold text indicates coverage lower than 90%, while italics indicates over‐coverage (higher than 97%).CCA, complete case analysis; SMI, single‐level imputation; FMI, fixed effect in the imputation model; MMI, multilevel MI; MI, multiple imputation; ICC,inter‐cluster correlation coefficient.

It is clear that the validity of inferences drawn depends crucially on the method chosen to handle the missing data. As the box and whisker plots for CI coverage show, the method that most consistently achieves coverage rates close to the nominal is MMI, as the interquartile range of the distribution of coverage across the 192 simulated scenarios is almost all contained within the limits 90– 97% (for example, only 8 scenarios out of the total 192 resulting in coverage for *Y*_1_ outside this range).

The results corresponding to RMSE are reported in the Supporting Information File 1. In general, MMI is more efficient than the other two MI methods. The ratio of MMI RMSE to either SMI or FMI RMSE is almost always ≤1, with only four scenarios resulting in a ratio >1.02. In general, the FMI RMSE is larger than those corresponding to the other two MI methods, in situations where the outcome ICCs were smaller than 0.2. Conversely, when ICCs are greater or equal than 0.2, the RMSE corresponding to SMI is larger than the corresponding RMSE for the other two methods.

## 5 Discussion

In this study, we compared the performance of single, multilevel and fixed‐effects MI for handling missing data in CRTs. The full‐factorial nature of our simulation study enabled us to establish which characteristics have the greatest influence on the performance of the alternative methods for handling missing data considered here.

In our simulations, which assumed the data were MAR throughout, bias was a serious problem for the CCA when the missingness mechanism was differential by treatment arm, while all MI methods resulted in unbiased treatment estimates. The main difference amongst the three MI procedures is in how variability is incorporated into the imputations. This is reflected in the variance estimates and has an impact on CI coverage rate. SMI resulted in low (<90*%*) coverage rate across most scenarios, in particular when the ICCs exceeded 0.05 and there were few clusters. Fixed‐effects MI produced overly conservative coverage (>98*%*), especially when there were small ICCs and more than 30 clusters. This finding reflects the way these two approaches accommodate the between‐cluster variance. Under SMI, the between‐cluster variance is set to zero, whereas with FMI, this variance is unbounded in the sense that the behaviour of one estimated cluster effect is unrelated, or unconstrained, by the behaviour of any of the others. Hence, FMI cannot be used to impute cluster‐level variables, or indeed, when the substantive model includes cluster‐level variables, because these cannot be explicitly included in the imputation model.

By contrast, MMI models the correlation in the data appropriately, producing coverage rates close to the nominal level. This consistent performance across the varying number of clusters and cluster sizes is indicative of acceptable finite sample properties. Moreover, MMI is compatible with the substantive model, in the sense that the imputation model contains the analysis model. The imputation model can include auxiliary variables at both the individual and the cluster‐level, thus increasing the plausibility of the MAR assumptions.

The re‐analysis of the OPERA study illustrates how each of the methods could be implemented in practice. In this re‐analysis, the standard errors for the estimated treatment effect for both outcomes are substantially larger when using FMI, while SMI resulted in smaller standard errors. From this and other simulation studies 24, 25, we know that FMI overestimates the variance, while SMI underestimates it. Moreover, there is a large difference in the estimates following the FMI, compared with other methods. This was larger for the endpoint (cost) where the ICC was smallest. This could be because the FMI cannot incorporate explicitly cluster size into the imputation model. In this example, the overall conclusion that the exercise intervention was not cost‐effective did not differ according to the approach taken to handling the missing data, but this may not always be the case.

The validity of the results when using MI depends on obtaining an appropriate estimate for the standard errors. This requires that the imputation model recognises the dependencies within the data, in this case amongst clusters. It is also important to use an appropriate number of imputations. A small *M* will translate into a loss of efficiency compared with the estimate obtained with infinitely many imputations. If we can accept a 5% loss of efficiency, then five imputations may be sufficient even for 25% missing information 4. In practice, the actual number of imputations necessary for MI to perform satisfactorily depends not only on the amount of information missing but also on the type of analysis. Some analyses may require *M* = 50 or more to obtain stable results 35. So, for a particular application, this number must be carefully chosen, based on sampling error of the MI estimates 10. In the present work, we used 10 imputations for the simulations and 50 for the illustrative example, following the recommendations in 10 for determining the required number of imputations.

We have shown how the flexibility of MMI allows the analyst to handle continuous multivariate outcomes without any modification to the multilevel imputation algorithm, because it is already based on multivariate normality. We illustrate this here using bivariate outcomes, but the generalisation to multivariate outcomes is straightforward. The MMI approach is readily available for continuous data in R packages pan 31 and jomo. The stand‐alone software RealcomImpute also performs MMI 36 and can be used in conjunction with Stata.

Previous simulation‐based comparisons of the alternative methods have been published before 24, 25. Our study builds on and extends the previous literature by establishing which characteristics of the setting most influence the performance of the different strategies for handling the missing data. In addition, we complement Andridge's work 25 by undertaking a more comprehensive assessment of the fixed effects MI, including complex scenarios with cluster‐level variables as predictors of missingness, varying cluster sizes and bivariate outcomes and showing further limitations of the FMI approach when compared with MMI.

The approach presented in this paper has some limitations. For simplicity, we assumed the missing data mechanism is MAR throughout. However, MI provides a flexible and convenient route for investigating sensitivity to alternative MNAR mechanisms (e.g. 37, Chapter 10]). Our simulations excluded situations with missing covariate data and where the imputation model is misspecified. MI assumes that the functional form of the imputation model has been correctly specified and includes all interactions and terms of higher order that are of substantive interest. A further concern could be that either the imputation or the analytical models make incorrect distributional assumptions. This was the case in the OPERA example, where we imputed the costs assuming a normal distribution. However, simulation studies by Schafer 13 and others 22, 34 have shown parametric MI to be fairly robust to misspecified distributions. Inferences are also insensitive to non‐Gaussian random effects in an multilevel imputation model, except when the rates of missingness are very high or the sample size is small 38.

Future research directions thus include considering MNAR mechanisms, especially those where the cluster random effect is driving the missingness. Other potential extensions relate to situations where there is cluster non‐response. In both situations, MMI could provide a flexible route for investigating sensitivity to alternative MNAR mechanisms and cluster dropout.

## Supporting information

Supporting info item
