# Supplementary material for: Multiple imputation methods for bivariate outcomes in cluster randomised trials
Source: Stat Med. 2016 Mar 14;35(20):3482–96. doi: 10.1002/sim.6935 (PMC4981911; doi:10.1002/sim.6935)
Supplement: Supplementary file 1 — Supporting info item [file SIM-35-3482-s001.zip › supplement_1.pdf]

Multiple imputation methods for bivariate outcomes in cluster randomised trials.

Supplementary File 1

Results for for Y_1

Table A1: Percentage bias for treatment effect on Y_1 for scenarios corresponding to missing mechanism associated with individual level covariate

Design	η	Prob. nonresponse	ICC	CCA	SMI	FMI	MMI
$J = 25$ $n_j = 10$	Low	.20,.20	0.01, 0.01	-1.3	-0.6	-0.7	-0.8
			0.20, 0.05	-1.3	-0.6	-0.6	-0.8
			0.20, 0.20	-1.3	-0.6	-0.6	-0.8
			0.60, 0.01	-1.2	-0.5	-0.4	-0.6
		.30,.10	0.01, 0.01	-1.4	-0.4	-0.9	-0.7
			0.20, 0.05	-1.4	-0.2	-0.8	-0.6
			0.20, 0.20	-1.4	-0.2	-0.8	-0.6
			0.60, 0.01	-1.3	0.3	-0.7	-0.5
	High	.20,.20	0.01, 0.01	-0.8	-0.8	-0.6	-0.6
			0.20, 0.05	-0.9	-0.8	-0.5	-0.5
			0.20, 0.20	-0.8	-0.8	-0.5	-0.5
			0.60, 0.01	-0.9	-0.8	-0.3	-0.3
		.30,.10	0.01, 0.01	-0.6	-0.7	-0.7	-0.5
			0.20, 0.05	-0.5	-0.3	-0.6	-0.3
			0.20, 0.20	-0.5	-0.3	-0.6	-0.3
			0.60, 0.01	-0.4	0.3	-0.4	-0.3
$J = 5$, $n_j = 50$	Low	.20,.20	0.01, 0.01	-0.6	-0.5	-0.3	-0.5
			0.20, 0.05	-2.4	-2.1	-2.0	-2.2
			0.20, 0.20	-2.4	-2.1	-2.0	-2.2
			0.60, 0.01	-5.6	-5.1	-5.1	-5.4
		.30,.10	0.01, 0.01	-1.1	-0.7	-1.0	-0.9
			0.20, 0.05	-2.9	-2.4	-2.8	-2.5
			0.20, 0.20	-2.9	-2.4	-2.8	-2.5
			0.60, 0.01	-0.9	0.7	-0.3	-0.1
	High	.20,.20	0.01, 0.01	-0.5	-0.6	-0.9	-1.0
			0.20, 0.05	-2.3	-2.2	-2.6	-2.7
			0.20, 0.20	-2.3	-2.3	-2.6	-2.7
			0.60, 0.01	-5.4	-4.9	-5.7	-5.9
		.30,.10	0.01, 0.01	-0.5	-0.3	0.0	-0.5
			0.20, 0.05	-2.3	-2.0	-1.7	-2.2
			0.20, 0.20	-2.3	-2.0	-1.7	-2.2
			0.60, 0.01	-5.5	-5.0	-4.8	-5.3
$J = 15$, unbalanced	Low	.20,.20	0.01, 0.01	0.6	0.3	0.4	0.3
			0.20, 0.05	-0.2	-0.6	-0.5	-0.5
			0.20, 0.20	-0.2	-0.6	-0.5	-0.5
			0.60, 0.01	-1.8	-2.2	-2.2	-2.1
		.30,.10	0.01, 0.01	0.8	0.2	0.2	0.3
			0.20, 0.05	-0.1	-0.7	-0.7	-0.6
			0.20, 0.20	-0.1	-0.8	-0.7	-0.7
			0.60, 0.01	-1.9	-2.4	-2.4	-2.4
	High	.20,.20	0.01, 0.01	0.4	-0.2	-0.1	-0.2
			0.20, 0.05	-0.5	-1.0	-1.0	-1.1
			0.20, 0.20	-0.5	-1.0	-0.9	-1.1
			0.60, 0.01	-2.1	-2.6	-2.6	-2.8
		.30,.10	0.01, 0.01	0.8	0.1	0.7	0.5
			0.20, 0.05	0.0	-0.8	-0.2	-0.2
			0.20, 0.20	-0.1	-0.9	-0.2	-0.3
			0.60, 0.01	-1.5	-2.6	-2.0	-1.9

Table A2: Percentage bias for treatment effect on Y_1 for scenarios corresponding to missing mechanism associated with cluster-level covariate

Design	η	Prob. nonresponse	ICC	CCA	SMI	FMI	MMI
$J = 25, n_j = 10$	Low	.20,.20	0.01, 0.01	-0.4	-0.9	-1.3	-0.7
			0.20, 0.05	-0.9	-1.0	-1.5	-1.0
			0.20, 0.20	-0.9	-1.1	-1.5	-1.0
			0.60, 0.01	-3.9	-2.9	-1.5	-1.9
		.30,.10	0.01, 0.01	-1.1	-1.1	-1.8	-1.4
			0.20, 0.05	-1.9	-0.9	-1.9	-1.5
			0.20, 0.20	-1.8	-0.9	-1.9	-1.4
			0.60, 0.01	-2.8	-0.6	-2.1	-2.0
	High	.20,.20	0.01, 0.01	-0.5	-0.6	-0.9	-0.3
			0.20, 0.05	-0.8	-0.5	-0.7	-0.3
			0.20, 0.20	-0.7	-0.5	-1.0	-0.2
			0.60, 0.01	-1.2	-0.4	-0.6	-0.9
		.30,.10	0.01, 0.01	-0.2	-0.3	-0.4	-0.3
			0.20, 0.05	-0.4	-0.2	-0.1	-0.3
			0.20, 0.20	-0.4	-0.2	-0.3	-0.3
			0.60, 0.01	-0.4	0.1	0.5	-0.9
$J = 5, n_j = 50$	Low	.20,.20	0.01, 0.01	0.0	0.1	0.1	0.1
			0.20, 0.05	-1.3	-0.9	-1.1	-0.8
			0.20, 0.20	-1.3	-0.9	-1.1	-0.8
			0.60, 0.01	-3.5	-2.9	-3.4	-2.6
		.30,.10	0.01, 0.01	-0.8	-0.3	-0.6	-0.3
			0.20, 0.05	-2.2	-1.2	-1.8	-1.2
			0.20, 0.20	-2.1	-1.2	-1.8	-1.1
			0.60, 0.01	-4.4	-2.7	-4.1	-3.1
	High	.20,.20	0.01, 0.01	0.0	-0.1	0.0	0.7
			0.20, 0.05	-1.8	-0.9	-1.3	-0.1
			0.20, 0.20	-1.9	-0.9	-0.7	0.1
			0.60, 0.01	-4.8	-2.5	-3.8	-1.8
		.30,.10	0.01, 0.01	-0.2	0.3	-0.1	0.5
			0.20, 0.05	-1.4	-0.2	0.0	0.2
			0.20, 0.20	-1.3	-0.1	-1.3	0.2
			0.60, 0.01	-4.1	-1.1	-3.1	-1.3
$J = 15$, unbalanced	Low	.20,.20	0.01, 0.01	0.6	0.3	0.6	0.6
			0.20, 0.05	0.1	-0.3	0.2	0.2
			0.20, 0.20	0.1	-0.3	0.2	0.2
			0.60, 0.01	-0.9	-1.6	-0.7	-0.8
		.30,.10	0.01, 0.01	0.2	0.6	0.7	0.5
			0.20, 0.05	-0.3	-0.2	0.3	0.1
			0.20, 0.20	-0.3	-0.2	0.2	0.0
			0.60, 0.01	-1.2	-1.7	-0.8	-1.0
	High	.20,.20	0.01, 0.01	-0.3	0.1	0.1	0.1
			0.20, 0.05	-1.1	-0.6	-0.1	-0.4
			0.20, 0.20	-1.0	-0.7	0.0	-0.5
			0.60, 0.01	-2.9	-2.2	-0.7	-0.9
		.30,.10	0.01, 0.01	-0.4	-0.3	0.1	-0.3
			0.20, 0.05	-1.9	-1.2	-0.7	-1.3
			0.20, 0.20	-1.8	-1.2	-0.5	-1.2
			0.60, 0.01	-3.9	-2.9	-1.5	-1.9

Table A3: Percentage bias for treatment effect on Y_1 for scenarios corresponding to missing mechanism dependent on individual and cluster-level covariates

Design	η	Prob. nonresponse	ICC	CCA	SMI	FMI	MMI
$J = 25, n_j = 10$	Low	.20,.20	0.01, 0.01	-0.6	-1.2	-1.4	-1.2
			0.20, 0.05	-1.1	-1.4	-1.6	-1.5
			0.20, 0.20	-1.0	-1.4	-1.7	-1.5
			0.60, 0.01	-1.7	-1.9	-2.0	-1.7
		.30,.10	0.01, 0.01	-1.2	-1.5	-1.8	-1.7
			0.20, 0.05	-2.0	-1.4	-1.9	-1.8
			0.20, 0.20	-1.9	-1.4	-1.8	-1.7
			0.60, 0.01	-2.9	-1.2	-2.1	-2.2
	High	.20,.20	0.01, 0.01	-0.4	-1.2	-1.8	-1.2
			0.20, 0.05	-1.1	-1.1	-1.9	-1.5
			0.20, 0.20	-1.2	-1.1	-2.0	-1.5
			0.60, 0.01	-2.4	-0.9	-2.2	-2.4
		.30,.10	0.01, 0.01	-0.6	-1.1	-0.4	-0.8
			0.20, 0.05	-1.0	-0.9	-0.5	-0.9
			0.20, 0.20	-1.0	-0.9	-0.3	-0.8
			0.60, 0.01	-1.3	-0.6	-0.5	-1.4
$J = 5, n_j = 50$	Low	.20,.20	0.01, 0.01	-0.2	0.1	0.2	0.1
			0.20, 0.05	-1.7	-1.0	-1.0	-0.8
			0.20, 0.20	-1.7	-1.0	-1.0	-0.7
			0.60, 0.01	-3.8	-3.1	-3.3	-2.6
		.30,.10	0.01, 0.01	-1.0	0.1	-0.3	-0.3
			0.20, 0.05	-2.7	-0.8	-1.6	-1.3
			0.20, 0.20	-2.7	-0.8	-1.5	-1.2
			0.60, 0.01	-4.8	-2.4	-3.8	-3.2
	High	.20,.20	0.01, 0.01	-0.9	0.7	-0.1	0.5
			0.20, 0.05	-2.4	0.1	-1.4	-0.3
			0.20, 0.20	-2.4	0.2	-1.3	-0.2
			0.60, 0.01	-4.7	-0.9	-2.8	-2.0
		.30,.10	0.01, 0.01	-0.3	-0.1	-0.2	0.0
			0.20, 0.05	-1.8	-0.6	-1.0	-0.7
			0.20, 0.20	-1.9	-0.5	-0.1	-0.6
			0.60, 0.01	-3.9	-1.9	-2.3	-2.7
$J = 15$, unbalanced	Low	.20,.20	0.01, 0.01	0.8	0.4	0.6	0.7
			0.20, 0.05	0.2	-0.2	0.1	0.3
			0.20, 0.20	0.1	-0.2	0.1	0.3
			0.60, 0.01	-1.0	-1.4	-0.9	-0.8
		.30,.10	0.01, 0.01	0.1	0.6	0.5	0.2
			0.20, 0.05	-0.5	-0.1	-0.1	-0.2
			0.20, 0.20	-0.6	-0.1	-0.1	-0.3
			0.60, 0.01	-1.6	-1.4	-1.3	-1.6
	High	.20,.20	0.01, 0.01	0.6	0.5	0.6	0.5
			0.20, 0.05	-0.2	0.0	0.1	0.0
			0.20, 0.20	-0.2	0.0	0.0	0.0
			0.60, 0.01	-1.6	-1.4	-1.3	-1.6
		.30,.10	0.01, 0.01	0.6	0.9	1.2	1.1
			0.20, 0.05	-0.2	0.0	0.5	0.6
			0.20, 0.20	-0.1	0.0	0.3	0.6
			0.60, 0.01	-1.3	-1.6	-0.6	-0.1

Table A4: Coverage rate and average width (AW) of the 95% CI for treatment effect on Y_1 after each of the MI strategies, when the missing mechanism depends only on individual level covariate

Design	η	Prob. Miss	ICC	CCA		SMI		FMI		MMI	
				CR	AW	CR	AW	CR	AW	CR	AW
$J = 25$, $n_j = 10$	Low	.20,.20	0.01, 0.01	95.1	18.4	94.5	16.7	97.8	19.7	95.3	16.9
			0.20, 0.05	94.2	28.1	91.8	24.0	96.5	29.2	94.4	26.8
			0.20, 0.20	93.9	28.2	91.6	24.0	96.6	29.2	93.7	26.4
			0.60, 0.01	94.3	56.5	87.8	46.4	95.1	57.2	94.3	56.0
		.30,.10	0.01, 0.01	95.3	18.5	95.6	17.9	98.2	22.6	95.0	18.1
			0.20, 0.05	93.5	28.3	89.5	23.9	96.6	31.3	93.8	27.3
			0.20, 0.20	93.8	28.3	89.8	23.8	96.5	31.3	92.7	26.6
			0.60, 0.01	95.2	56.7	85.3	44.0	95.2	58.2	95.0	56.4
	High	.20,.20	0.01, 0.01	94.4	18.5	94.0	18.3	97.7	22.6	94.6	18.2
			0.20, 0.05	93.8	28.3	89.9	24.7	96.8	31.3	93.7	27.6
			0.20, 0.20	93.8	28.3	89.8	24.7	96.8	31.3	93.1	27.1
			0.60, 0.01	94.2	56.7	87.4	45.7	95.2	58.3	94.0	56.5
		.30,.10	0.01, 0.01	94.6	18.6	96.3	20.3	97.8	25.5	95.0	19.8
			0.20, 0.05	93.4	28.4	91.7	25.8	96.8	33.4	93.2	28.4
			0.20, 0.20	93.6	28.4	91.7	25.7	97.0	33.4	92.6	27.8
			0.60, 0.01	94.2	56.8	86.2	45.3	95.4	59.5	94.3	57.1
$J = 5$, $n_j = 50$	Low	.20,.20	0.01, 0.01	93.9	20.3	93.0	18.1	96.0	20.9	92.8	18.5
			0.20, 0.05	89.7	50.9	83.2	40.4	91.1	52.0	89.6	50.1
			0.20, 0.20	89.6	50.7	83.3	40.8	91.5	52.3	90.0	50.0
			0.60, 0.01	90.8	118.7	81.9	91.9	90.6	120.1	90.2	118.8
		.30,.10	0.01, 0.01	94.4	20.4	92.8	19.2	96.5	23.0	92.9	19.3
			0.20, 0.05	89.8	51.1	78.6	37.4	92.3	53.0	89.3	49.8
			0.20, 0.20	90.4	50.8	79.1	37.8	92.5	53.2	89.7	49.7
			0.60, 0.01	92.1	120.2	79.6	82.9	92.9	121.0	92.2	119.7
	High	.20,.20	0.01, 0.01	93.3	20.4	92.4	19.4	96.2	23.0	93.2	20.1
			0.20, 0.05	90.1	51.4	80.8	38.7	92.3	53.0	89.7	50.4
			0.20, 0.20	90.2	51.3	81.3	39.0	92.1	53.1	89.5	50.2
			0.60, 0.01	90.4	119.4	79.0	85.9	91.1	120.7	90.4	119.0
		.30,.10	0.01, 0.01	94.1	20.4	93.3	21.2	96.7	25.2	93.0	21.6
			0.20, 0.05	89.8	51.0	78.3	37.1	93.0	54.0	89.7	50.4
			0.20, 0.20	89.5	51.1	78.7	37.5	93.1	54.2	89.5	50.1
			0.60, 0.01	89.9	118.9	75.0	79.2	91.7	120.8	91.8	119.2
$J = 15$, unbalanced	Low	.20,.20	0.01, 0.01	94.5	17.5	93.4	15.8	97.2	18.9	93.2	16.0
			0.20, 0.05	93.6	33.2	88.3	27.5	95.7	34.5	92.9	32.2
			0.20, 0.20	93.6	33.2	88.3	27.5	95.7	34.5	92.9	32.2
			0.60, 0.01	93.9	71.6	85.8	57.3	94.7	72.4	94.5	71.2
		.30,.10	0.01, 0.01	93.2	17.7	93.9	17.1	97.4	21.7	92.9	17.0
			0.20, 0.05	92.8	33.4	85.4	26.6	96.3	36.2	92.3	32.4
			0.20, 0.20	93.5	33.4	85.3	26.6	96.4	36.1	91.9	32.1
			0.60, 0.01	93.6	71.5	82.5	53.1	94.9	73.2	94.1	71.5
	High	.20,.20	0.01, 0.01	93.4	17.7	92.8	17.4	97.2	21.3	93.4	17.5
			0.20, 0.05	92.6	33.4	86.0	27.4	95.7	35.9	93.4	32.9
			0.20, 0.20	93.0	33.4	86.1	27.4	95.7	35.9	93.4	32.6
			0.60, 0.01	93.9	71.7	83.4	55.1	94.8	73.2	93.8	71.6
		.30,.10	0.01, 0.01	93.9	17.8	93.7	19.0	98.0	24.4	93.7	18.9
			0.20, 0.05	93.8	33.5	84.8	27.4	97.0	37.9	93.7	33.5
			0.20, 0.20	94.0	33.5	84.4	27.3	96.8	37.8	93.6	33.1
			0.60, 0.01	94.3	71.6	80.3	52.5	94.3	74.1	93.9	72.0

Table A5: Coverage rate and average width (AW) of the 95 CI for treatment effect on Y_1 after each of the MI strategies, when the missing mechanism depends only on cluster level covariate

Design	η	Prob. Miss	ICC	CCA		SMI		FMI		MMI	
				CR	AW	CR	AW	CR	AW	CR	AW
$J = 25$	Low	.20,.20	0.01, 0.01	93.9	19.3	95.4	18.0	98.0	23.5	95.5	18.0
$n_j = 10$			0.20, 0.05	94.8	29.0	91.7	25.9	97.0	32.2	93.4	27.4
			0.20, 0.20	94.9	29.1	91.7	25.9	97.1	32.2	93.2	27.2
			0.60, 0.01	92.9	73.8	85.6	63.1	93.7	79.2	93.7	75.9
		.30,.10	0.01, 0.01	94.9	19.4	94.6	19.6	99.1	27.8	94.6	19.5
			0.20, 0.05	95.3	29.2	91.9	26.5	97.6	35.6	92.8	28.1
			0.20, 0.20	95.2	29.1	92.2	26.6	97.8	35.5	92.6	27.9
			0.60, 0.01	94.4	57.7	89.4	49.4	95.4	61.1	94.2	57.7
	High	.20,.20	0.01, 0.01	93.9	19.8	95.1	20.2	99.3	29.6	95.7	20.2
			0.20, 0.05	93.6	29.9	92.5	27.6	97.6	36.7	94.3	29.2
			0.20, 0.20	92.8	29.8	92.5	27.6	97.8	37.0	93.9	29.0
			0.60, 0.01	94.6	59.2	89.0	51.6	96.4	61.5	94.8	59.2
		.30,.10	0.01, 0.01	95.3	19.9	95.2	22.4	99.1	33.9	94.4	21.8
			0.20, 0.05	93.5	29.9	91.3	29.1	98.3	40.3	93.3	30.4
			0.20, 0.20	93.9	30.0	91.4	29.1	98.4	40.7	93.3	30.4
			0.60, 0.01	94.9	59.2	88.5	52.0	95.6	63.8	95.1	60.5
$J = 5$	Low	.20,.20	0.01, 0.01	94.4	21.2	95.3	20.5	97.5	23.6	96.2	20.8
$n_j = 50$			0.20, 0.05	91.1	51.6	89.2	46.4	92.9	53.7	91.7	51.0
			0.20, 0.20	90.8	51.7	89.0	46.4	92.8	53.6	91.5	51.1
			0.60, 0.01	91.0	119.2	86.7	105.7	91.5	122.1	91.1	120.2
		.30,.10	0.01, 0.01	92.9	21.3	95.3	22.4	98.1	26.9	95.8	22.5
			0.20, 0.05	90.1	51.7	87.8	45.9	93.9	55.4	91.1	51.1
			0.20, 0.20	89.9	51.7	87.6	46.0	93.6	55.4	91.5	51.2
			0.60, 0.01	91.2	119.3	85.1	101.8	91.6	122.3	90.8	120.4
	High	.20,.20	0.01, 0.01	94.4	21.9	96.3	24.1	98.9	32.3	96.6	29.1
			0.20, 0.05	89.7	52.0	87.7	48.4	94.1	60.0	90.7	58.5
			0.20, 0.20	89.1	52.3	88.1	48.5	93.8	61.9	91.6	58.6
			0.60, 0.01	89.9	120.1	85.0	107.6	91.8	128.2	91.9	128.7
		.30,.10	0.01, 0.01	93.9	21.9	96.8	27.1	98.4	38.7	96.7	29.9
			0.20, 0.05	89.7	52.3	88.8	49.6	95.3	65.4	91.3	57.5
			0.20, 0.20	89.9	52.3	89.2	49.5	95.3	65.5	91.8	57.7
			0.60, 0.01	89.8	119.8	84.1	106.2	92.3	129.4	91.1	127.6
$J = 15$	Low	.20,.20	0.01, 0.01	93.6	18.3	95.1	17.4	97.9	22.4	95.2	17.4
unbalanced			0.20, 0.05	91.9	34.0	89.0	30.0	95.5	37.0	92.6	32.8
			0.20, 0.20	91.9	34.1	88.8	30.0	95.4	36.9	92.1	32.5
			0.60, 0.01	92.6	72.7	87.5	62.8	94.4	74.8	93.8	72.6
		.30,.10	0.01, 0.01	95.3	18.3	95.9	18.8	99.0	26.5	95.4	18.7
			0.20, 0.05	93.6	34.0	88.7	30.0	96.7	39.6	92.4	33.2
			0.20, 0.20	93.1	34.0	88.8	30.1	96.7	39.6	92.1	32.8
			0.60, 0.01	93.5	72.6	85.6	60.7	94.9	76.0	93.7	73.0
	High	.20,.20	0.01, 0.01	93.7	18.9	94.6	19.4	99.1	29.9	94.5	19.5
			0.20, 0.05	91.6	34.8	88.1	31.3	96.2	42.2	92.2	34.4
			0.20, 0.20	91.2	34.8	87.8	31.3	96.2	42.2	92.3	34.0
			0.60, 0.01	92.6	73.9	86.5	63.6	94.4	77.0	93.6	74.4
		.30,.10	0.01, 0.01	93.7	18.9	95.9	21.8	99.6	35.3	94.8	21.6
			0.20, 0.05	91.9	34.8	87.8	32.4	96.9	45.3	92.0	35.6
			0.20, 0.20	91.4	34.8	88.2	32.4	97.1	45.9	91.9	35.3
			0.60, 0.01	92.9	73.8	85.6	63.1	93.7	79.2	93.7	75.9

Table A6: Coverage rate and average width (AW) of the 95 CI for treatment effect on Y_1 after each of the MI strategies, when the missing mechanism depends individual and cluster-level covariates

Design	η	Prob. Miss	ICC	CCA		SMI		FMI		MMI	
				CR	AW	CR	AW	CR	AW	CR	AW
$J = 25$, $n_j = 50$	Low	.20,.20	0.01, 0.01	95.2	18.8	95.1	17.7	98.7	22.1	95.2	17.6
			0.20, 0.05	94.1	27.5	91.5	25.7	96.9	31.1	93.5	27.5
			0.20, 0.20	94.0	27.6	91.3	25.8	96.7	31.1	93.1	27.2
			0.60, 0.01	94.6	55.8	90.1	50.3	95.4	58.7	94.5	57.0
		.30,.10	0.01, 0.01	95.1	18.9	96.1	19.5	99.1	25.9	95.8	19.0
			0.20, 0.05	94.3	27.7	90.9	26.6	97.8	33.8	93.3	28.2
			0.20, 0.20	94.9	27.7	90.8	26.7	97.9	33.9	92.8	27.8
			0.60, 0.01	94.3	55.9	89.6	49.8	95.8	60.1	94.4	57.5
	High	.20,.20	0.01, 0.01	95.1	19.3	96.5	20.7	98.8	27.8	95.7	20.6
			0.20, 0.05	94.2	27.5	93.2	28.0	98.1	35.4	94.3	29.7
			0.20, 0.20	93.7	27.5	93.0	28.0	98.1	35.3	94.2	29.5
			0.60, 0.01	94.8	55.3	90.2	51.7	96.3	61.0	94.8	58.7
		.30,.10	0.01, 0.01	94.8	19.3	96.3	22.8	99.0	31.6	95.4	22.3
			0.20, 0.05	93.7	27.5	92.9	29.5	98.6	38.2	94.9	30.9
			0.20, 0.20	94.0	27.4	92.9	29.6	98.5	38.4	94.2	30.6
			0.60, 0.01	94.9	55.4	88.2	52.5	95.7	62.3	94.8	59.7
$J = 5$ $n_j = 50$	Low	.20,.20	0.01, 0.01	94.3	20.5	95.4	20.2	97.0	22.4	95.1	20.3
			0.20, 0.05	89.7	47.5	88.0	46.3	92.3	52.9	90.4	51.2
			0.20, 0.20	89.5	47.8	87.6	46.3	92.0	52.8	90.4	51.1
			0.60, 0.01	90.4	115.3	86.6	105.5	91.1	121.2	90.7	120.3
		.30,.10	0.01, 0.01	94.2	20.5	95.5	21.9	97.7	25.5	95.7	21.8
			0.20, 0.05	89.7	47.8	86.7	45.7	92.4	54.5	90.6	51.3
			0.20, 0.20	89.3	48.0	87.1	45.8	92.5	54.4	90.7	51.3
			0.60, 0.01	90.4	115.4	84.9	101.8	91.2	121.7	90.7	120.3
	High	.20,.20	0.01, 0.01	92.9	22.6	96.1	23.7	98.0	29.8	95.8	23.8
			0.20, 0.05	88.2	46.4	86.7	47.4	92.9	57.4	90.4	52.9
			0.20, 0.20	89.1	46.3	86.9	47.4	93.4	57.5	90.9	52.9
			0.60, 0.01	90.6	112.8	85.1	104.7	91.6	126.3	90.9	122.2
		.30,.10	0.01, 0.01	91.7	22.7	97.0	26.4	98.8	33.7	97.4	26.3
			0.20, 0.05	89.3	46.6	87.6	48.1	93.6	59.1	91.0	53.8
			0.20, 0.20	89.3	46.5	87.4	48.1	93.7	62.2	90.8	53.7
			0.60, 0.01	90.7	112.4	83.5	103.2	91.9	126.5	91.6	122.7
$J = 15$, unbalanced	Low	.20,.20	0.01, 0.01	93.6	17.9	95.6	17.0	98.1	21.0	94.8	16.9
			0.20, 0.05	93.0	31.9	89.8	29.9	95.4	36.1	92.5	32.9
			0.20, 0.20	92.8	32.0	89.9	29.9	95.4	36.0	92.1	32.6
			0.60, 0.01	92.9	70.2	87.9	62.7	94.3	74.0	93.9	72.4
		.30,.10	0.01, 0.01	93.9	17.9	95.4	18.4	98.6	24.7	94.6	18.2
			0.20, 0.05	93.3	31.9	88.7	29.9	96.1	38.4	92.5	33.3
			0.20, 0.20	92.8	31.9	88.3	29.9	96.1	38.3	92.1	32.9
			0.60, 0.01	94.2	70.1	86.1	60.8	94.4	75.0	93.3	72.7
	High	.20,.20	0.01, 0.01	94.9	18.8	95.1	19.5	98.7	27.3	94.9	19.7
			0.20, 0.05	92.3	31.6	89.3	31.0	96.8	40.1	94.3	34.6
			0.20, 0.20	92.8	31.6	89.0	31.0	96.8	40.2	93.9	34.3
			0.60, 0.01	94.2	70.1	86.1	60.8	94.4	75.0	93.3	72.7
		.30,.10	0.01, 0.01	93.7	18.8	94.2	21.4	99.0	31.1	94.8	21.4
			0.20, 0.05	92.6	31.6	87.9	31.9	96.8	42.7	92.8	35.6
			0.20, 0.20	92.3	31.6	88.1	31.9	96.8	43.1	92.3	35.1
			0.60, 0.01	93.4	69.2	84.3	62.1	94.2	77.5	94.0	74.7

Table A7: RMSE for treatment estimate on Y_1 after each of the MI methods under comparison, for scenarios corresponding to missing data mechanisms associated with individual covariate

Design	η	Prob. nonresponse	ICC	CCA	SMI	FMI	MMI
$J = 25$, $n_j = 10$	Low	.20,.20	0.01, 0.01	4.67	4.20	4.24	4.20
			0.20, 0.05	7.20	6.93	6.86	6.88
			0.20, 0.20	7.20	6.93	6.87	6.89
			0.60, 0.01	14.41	14.48	14.21	14.25
		.30,.10	0.01, 0.01	4.62	4.38	4.53	4.35
			0.20, 0.05	7.20	7.11	7.07	7.04
			0.20, 0.20	7.20	7.11	7.07	7.06
			0.60, 0.01	14.44	14.72	14.34	14.35
	High	.20,.20	0.01, 0.01	4.77	4.59	4.69	4.67
			0.20, 0.05	7.31	7.31	7.15	7.25
			0.20, 0.20	7.32	7.31	7.15	7.28
			0.60, 0.01	14.50	15.02	14.35	14.42
		.30,.10	0.01, 0.01	4.76	4.90	5.18	4.99
			0.20, 0.05	7.35	7.61	7.48	7.52
			0.20, 0.20	7.35	7.62	7.48	7.53
			0.60, 0.01	14.56	15.38	14.52	14.55
$J = 5$, $n_j = 50$	Low	.20,.20	0.01, 0.01	5.29	4.98	4.96	5.03
			0.20, 0.05	13.46	13.46	13.40	13.41
			0.20, 0.20	13.46	13.46	13.40	13.42
			0.60, 0.01	31.40	31.57	31.44	31.44
		.30,.10	0.01, 0.01	5.24	5.20	5.20	5.20
			0.20, 0.05	13.47	13.58	13.46	13.46
			0.20, 0.20	13.48	13.57	13.46	13.46
			0.60, 0.01	31.60	31.80	31.57	31.57
	High	.20,.20	0.01, 0.01	5.33	5.26	5.32	5.28
			0.20, 0.05	13.51	13.58	13.55	13.56
			0.20, 0.20	13.51	13.58	13.55	13.56
			0.60, 0.01	31.46	31.65	31.52	31.53
		.30,.10	0.01, 0.01	5.39	5.69	5.71	5.69
			0.20, 0.05	13.60	13.75	13.65	13.66
			0.20, 0.20	13.60	13.76	13.65	13.66
			0.60, 0.01	31.53	31.78	31.51	31.52
$J = 15$, unbalanced	Low	.20,.20	0.01, 0.01	4.55	4.27	4.29	4.25
			0.20, 0.05	8.89	8.74	8.62	8.64
			0.20, 0.20	8.89	8.74	8.62	8.64
			0.60, 0.01	19.00	19.09	18.77	18.79
		.30,.10	0.01, 0.01	4.77	4.56	4.60	4.55
			0.20, 0.05	9.09	9.05	8.87	8.87
			0.20, 0.20	9.09	9.05	8.86	8.88
			0.60, 0.01	19.16	19.48	18.97	18.99
	High	.20,.20	0.01, 0.01	4.60	4.64	4.67	4.64
			0.20, 0.05	8.90	9.14	8.84	8.92
			0.20, 0.20	8.90	9.14	8.83	8.92
			0.60, 0.01	18.98	19.65	18.91	18.98
		.30,.10	0.01, 0.01	4.68	4.95	5.03	5.01
			0.20, 0.05	8.94	9.35	9.00	9.02
			0.20, 0.20	8.95	9.36	9.00	9.04
			0.60, 0.01	18.97	19.92	18.96	18.91

Table A8: RMSE for treatment estimate on Y_1 after each of the MI methods under comparison, for scenarios corresponding to missing data mechanisms associated with cluster-level covariate

Design	η	Prob. nonresponse	ICC	CCA	SMI	FMI	MMI
$J = 25, n_j = 10$	Low	.20,.20	0.01, 0.01	4.92	4.42	4.57	4.44
			0.20, 0.05	7.47	7.16	7.22	7.25
			0.20, 0.20	7.47	7.16	7.22	7.25
			0.60, 0.01	19.61	21.34	20.46	20.12
		.30,.10	0.01, 0.01	4.88	4.78	5.19	4.83
			0.20, 0.05	7.43	7.42	7.64	7.45
			0.20, 0.20	7.41	7.43	7.64	7.45
			0.60, 0.01	14.64	15.06	14.82	14.80
	High	.20,.20	0.01, 0.01	5.09	5.00	5.44	4.99
			0.20, 0.05	7.81	7.76	7.68	7.64
			0.20, 0.20	7.81	7.75	7.69	7.64
			0.60, 0.01	15.24	15.71	14.76	14.88
		.30,.10	0.01, 0.01	5.01	5.30	5.92	5.31
			0.20, 0.05	7.71	8.16	8.33	7.98
			0.20, 0.20	7.72	8.16	8.33	7.99
			0.60, 0.01	15.19	16.35	15.62	15.24
$J = 5, n_j = 50$	Low	.20,.20	0.01, 0.01	5.41	5.14	5.21	5.06
			0.20, 0.05	13.34	13.42	13.34	13.24
			0.20, 0.20	13.36	13.42	13.34	13.23
			0.60, 0.01	31.16	31.53	31.26	31.16
		.30,.10	0.01, 0.01	5.54	5.51	5.51	5.51
			0.20, 0.05	13.43	13.62	13.43	13.44
			0.20, 0.20	13.44	13.60	13.42	13.46
			0.60, 0.01	31.23	31.75	31.27	31.27
	High	.20,.20	0.01, 0.01	5.55	5.65	5.99	7.41
			0.20, 0.05	13.64	14.17	13.82	14.83
			0.20, 0.20	13.64	14.14	14.39	14.82
			0.60, 0.01	31.22	33.05	31.79	32.49
		.30,.10	0.01, 0.01	5.68	6.30	6.85	6.43
			0.20, 0.05	13.82	14.50	14.88	14.29
			0.20, 0.20	13.83	14.47	14.36	14.28
			0.60, 0.01	31.42	33.49	31.95	32.19
$J = 15$, unbalanced	Low	.20,.20	0.01, 0.01	4.79	4.44	4.59	4.39
			0.20, 0.05	9.12	9.04	9.00	8.87
			0.20, 0.20	9.12	9.03	9.00	8.88
			0.60, 0.01	19.35	19.64	19.30	19.15
		.30,.10	0.01, 0.01	4.69	4.65	5.10	4.70
			0.20, 0.05	9.04	9.21	9.28	9.16
			0.20, 0.20	9.05	9.20	9.28	9.16
			0.60, 0.01	19.28	19.86	19.46	19.47
	High	.20,.20	0.01, 0.01	4.89	4.91	5.55	4.99
			0.20, 0.05	9.49	9.73	9.74	9.57
			0.20, 0.20	9.50	9.72	9.74	9.59
			0.60, 0.01	19.74	20.78	20.11	19.79
		.30,.10	0.01, 0.01	4.93	5.37	6.27	5.44
			0.20, 0.05	9.42	10.08	10.11	9.83
			0.20, 0.20	9.43	10.06	10.15	9.87
			0.60, 0.01	19.61	21.34	20.46	20.12

Table A9: RMSE for treatment estimate on Y_1 after each of the MI methods under comparison, for scenarios corresponding to missing data mechanisms associated with both individual and cluster-level covariate

Design	η	Prob. nonresponse	ICC	CCA	SMI	FMI	MMI
$J = 25, n_j = 10$	Low	.20,.20	0.01, 0.01	4.76	4.27	4.37	4.33
			0.20, 0.05	7.07	7.11	7.08	7.16
			0.20, 0.20	7.07	7.12	7.08	7.16
			0.60, 0.01	14.20	14.81	14.52	14.65
		.30,.10	0.01, 0.01	4.68	4.67	5.08	4.71
			0.20, 0.05	7.07	7.36	7.48	7.35
			0.20, 0.20	7.07	7.36	7.49	7.37
			0.60, 0.01	14.20	15.08	14.67	14.74
	High	.20,.20	0.01, 0.01	4.92	4.87	5.26	4.88
			0.20, 0.05	7.14	7.65	7.54	7.47
			0.20, 0.20	7.14	7.65	7.55	7.49
			0.60, 0.01	14.25	15.67	14.58	14.74
		.30,.10	0.01, 0.01	4.88	5.22	5.70	5.30
			0.20, 0.05	7.10	7.96	8.02	7.91
			0.20, 0.20	7.11	7.96	8.04	7.96
			0.60, 0.01	14.07	16.05	15.13	15.14
$J = 25, n_j = 10$	Low	.20,.20	0.01, 0.01	5.32	5.06	5.08	5.00
			0.20, 0.05	12.54	13.47	13.37	13.31
			0.20, 0.20	12.56	13.47	13.37	13.31
			0.60, 0.01	30.15	31.62	31.33	31.26
		.30,.10	0.01, 0.01	5.33	5.46	5.47	5.37
			0.20, 0.05	12.62	13.77	13.54	13.45
			0.20, 0.20	12.60	13.76	13.54	13.45
			0.60, 0.01	30.24	31.99	31.42	31.33
	High	.20,.20	0.01, 0.01	6.14	5.78	6.06	5.78
			0.20, 0.05	12.71	14.13	14.05	13.88
			0.20, 0.20	12.74	14.13	13.99	13.89
			0.60, 0.01	29.71	32.58	32.46	31.71
		.30,.10	0.01, 0.01	6.02	6.03	6.57	6.03
			0.20, 0.05	12.48	14.18	13.92	13.98
			0.20, 0.20	12.50	14.19	14.65	13.97
			0.60, 0.01	29.53	32.70	31.75	31.96
$J = 15$, unbalanced	Low	.20,.20	0.01, 0.01	4.64	4.32	4.51	4.35
			0.20, 0.05	8.51	8.92	8.88	8.87
			0.20, 0.20	8.52	8.94	8.88	8.89
			0.60, 0.01	18.55	19.53	19.16	19.16
		.30,.10	0.01, 0.01	4.70	4.62	5.00	4.63
			0.20, 0.05	8.60	9.19	9.21	9.05
			0.20, 0.20	8.60	9.19	9.21	9.05
			0.60, 0.01	18.62	19.88	19.39	19.32
	High	.20,.20	0.01, 0.01	4.90	4.94	5.43	4.93
			0.20, 0.05	8.48	9.58	9.61	9.35
			0.20, 0.20	8.51	9.59	9.64	9.35
			0.60, 0.01	18.62	19.88	19.39	19.32
		.30,.10	0.01, 0.01	4.92	5.45	5.88	5.40
			0.20, 0.05	8.52	10.03	9.73	9.67
			0.20, 0.20	8.54	10.03	9.75	9.71
			0.60, 0.01	18.41	21.20	19.87	19.75

Table A10: RMSE for treatment estimate on Y_1 after each of the MI methods under comparison, for scenarios corresponding to missing data mechanisms differential by treatment arm.

Design	η	Prob. nonresponse	ICC	CCA	SMI	FMI	MMI
$J = 25$, $n_j = 10$	Low	.20,.20	0.01, 0.01	6.86	4.37	4.63	4.39
			0.20, 0.05	9.69	7.14	7.25	7.16
			0.20, 0.20	9.72	7.14	7.25	7.16
			0.60, 0.01	16.17	14.77	14.63	14.64
		.30,.10	0.01, 0.01	6.64	4.74	4.96	4.71
			0.20, 0.05	9.48	7.47	7.49	7.45
			0.20, 0.20	9.49	7.47	7.49	7.48
			0.60, 0.01	16.06	15.11	14.77	14.76
	High	.20,.20	0.01, 0.01	7.15	4.32	4.46	4.30
			0.20, 0.05	10.12	7.24	7.15	7.09
			0.20, 0.20	10.17	7.24	7.15	7.10
			0.60, 0.01	16.46	15.02	14.60	14.60
		.30,.10	0.01, 0.01	7.36	4.42	4.65	4.46
			0.20, 0.05	10.44	7.32	7.25	7.26
			0.20, 0.20	10.51	7.33	7.26	7.27
			0.60, 0.01	16.90	15.16	14.60	14.65
$J = 5$, $n_j = 50$	Low	.20,.20	0.01, 0.01	7.40	5.20	5.20	5.16
			0.20, 0.05	14.35	13.70	13.56	13.50
			0.20, 0.20	14.41	13.69	13.56	13.50
			0.60, 0.01	31.34	31.87	31.53	31.45
		.30,.10	0.01, 0.01	7.29	5.37	5.45	5.32
			0.20, 0.05	14.11	13.71	13.65	13.54
			0.20, 0.20	14.16	13.71	13.65	13.54
			0.60, 0.01	31.00	31.84	31.55	31.46
	High	.20,.20	0.01, 0.01	7.77	5.07	5.10	5.05
			0.20, 0.05	14.57	13.62	13.43	13.42
			0.20, 0.20	14.61	13.62	13.42	13.44
			0.60, 0.01	31.25	31.87	31.39	31.37
		.30,.10	0.01, 0.01	8.19	5.32	8.96	5.29
			0.20, 0.05	14.86	13.83	13.61	13.68
			0.20, 0.20	14.93	13.82	13.67	13.67
			0.60, 0.01	31.36	32.13	31.51	31.77
$J = 15$, unbalanced	Low	.20,.20	0.01, 0.01	6.59	4.41	4.55	4.45
			0.20, 0.05	10.49	8.95	8.88	8.86
			0.20, 0.20	10.54	8.95	8.87	8.87
			0.60, 0.01	19.87	19.46	19.15	19.13
		.30,.10	0.01, 0.01	6.54	4.77	5.03	4.82
			0.20, 0.05	10.47	9.27	9.19	9.11
			0.20, 0.20	10.50	9.27	9.20	9.13
			0.60, 0.01	19.84	19.87	19.40	19.23
	High	.20,.20	0.01, 0.01	6.92	4.36	4.48	4.39
			0.20, 0.05	10.86	8.95	8.84	8.89
			0.20, 0.20	10.93	8.95	8.84	8.89
			0.60, 0.01	20.01	19.55	19.17	19.15
		.30,.10	0.01, 0.01	7.24	4.59	4.95	4.60
			0.20, 0.05	11.25	9.08	9.18	9.03
			0.20, 0.20	11.31	9.08	9.17	9.04
			0.60, 0.01	20.39	19.66	19.45	19.34

Results for Y_2

Table A11: Percentage bias for treatment effect on Y_2 for scenarios corresponding to missing mechanism associated with individual level covariate

Design	η	Prob. nonresponse	ICC	CCA	SMI	FMI	MMI
$J = 25, n_j = 10$	Low	.20,.20	0.01, 0.01	-1.62	-1.46	-1.45	-1.55
			0.20, 0.05	-1.75	-1.57	-1.60	-1.69
			0.20, 0.20	-1.98	-1.86	-1.92	-2.05
			0.60, 0.01	-1.61	-1.42	-1.45	-1.53
		.30,.10	0.01, 0.01	-1.49	-1.60	-1.41	-1.60
			0.20, 0.05	-1.58	-1.73	-1.56	-1.71
			0.20, 0.20	-1.80	-2.02	-1.88	-2.00
			0.60, 0.01	-1.47	-1.57	-1.41	-1.61
	High	.20,.20	0.01, 0.01	-0.69	-1.84	-2.23	-1.82
			0.20, 0.05	-0.86	-2.05	-2.38	-2.02
			0.20, 0.20	-1.03	-2.48	-2.69	-2.34
			0.60, 0.01	-0.69	-1.85	-2.22	-1.81
		.30,.10	0.01, 0.01	-0.49	-1.72	-1.43	-1.46
			0.20, 0.05	-0.68	-1.82	-1.58	-1.57
			0.20, 0.20	-0.97	-2.06	-1.90	-1.91
			0.60, 0.01	-0.44	-1.68	-1.43	-1.44
$J = 5, n_j = 50$	Low	.20,.20	0.01, 0.01	-0.74	-0.01	-0.25	-0.26
			0.20, 0.05	-1.51	-0.71	-0.97	-0.98
			0.20, 0.20	-3.03	-2.19	-2.47	-2.48
			0.60, 0.01	-0.75	0.00	-0.26	-0.25
		.30,.10	0.01, 0.01	-1.33	-0.42	-0.43	-0.37
			0.20, 0.05	-2.05	-1.13	-1.14	-1.09
			0.20, 0.20	-3.52	-2.63	-2.64	-2.60
			0.60, 0.01	-1.59	-1.27	-1.28	-1.19
	High	.20,.20	0.01, 0.01	-0.59	-0.83	-0.77	-0.98
			0.20, 0.05	-1.28	-1.50	-1.48	-1.68
			0.20, 0.20	-2.79	-2.97	-2.99	-3.16
			0.60, 0.01	-0.57	-0.80	-0.75	-0.97
		.30,.10	0.01, 0.01	-0.54	-0.22	-0.28	-0.17
			0.20, 0.05	-1.29	-0.94	-0.99	-0.88
			0.20, 0.20	-2.82	-2.48	-2.49	-2.37
			0.60, 0.01	-0.55	-0.21	-0.27	-0.16
$J = 15$, unbalanced	Low	.20,.20	0.01, 0.01	-0.43	0.38	0.20	0.37
			0.20, 0.05	-1.24	-0.37	-0.57	-0.42
			0.20, 0.20	-1.24	-0.37	-0.57	-0.42
			0.60, 0.01	-0.46	0.39	0.16	0.32
		.30,.10	0.01, 0.01	0.03	-0.01	0.03	-0.03
			0.20, 0.05	-0.60	-0.69	-0.70	-0.77
			0.20, 0.20	-2.13	-2.25	-2.21	-2.31
			0.60, 0.01	0.00	0.00	-0.03	-0.08
	High	.20,.20	0.01, 0.01	-1.03	-0.21	0.02	-0.14
			0.20, 0.05	-1.65	-0.95	-0.65	-0.89
			0.20, 0.20	-3.12	-2.61	-2.03	-2.23
			0.60, 0.01	-1.01	-0.21	-0.02	-0.20
		.30,.10	0.01, 0.01	-0.05	0.28	0.58	0.20
			0.20, 0.05	-0.68	-0.44	-0.17	-0.57
			0.20, 0.20	-2.17	-2.05	-1.72	-2.06
			0.60, 0.01	-0.01	0.28	0.53	0.17

Table A12: Percentage bias for treatment effect on Y_2 for scenarios corresponding to missing mechanism depending on cluster-level covariate

Design	η	Prob. nonresponse	ICC	CCA	SMI	FMI	MMI
$J = 25, n_j = 10$	Low	.20,.20	0.01, 0.01	-1.53	-1.88	-1.44	-1.70
			0.20, 0.05	-1.79	-2.13	-1.65	-1.93
			0.20, 0.20	-2.29	-2.66	-2.11	-2.39
			0.60, 0.01	-1.06	-0.51	-0.65	-0.63
		.30,.10	0.01, 0.01	-0.49	-0.95	-1.00	-0.90
			0.20, 0.05	-0.86	-1.18	-1.21	-1.11
			0.20, 0.20	-1.38	-1.68	-1.66	-1.55
			0.60, 0.01	-0.47	-0.95	-0.99	-0.88
	High	.20,.20	0.01, 0.01	-1.12	-1.56	-1.34	-1.59
			0.20, 0.05	-1.43	-1.80	-1.59	-1.81
			0.20, 0.20	-1.75	-2.28	-2.17	-2.08
			0.60, 0.01	-1.13	-1.60	-1.54	-1.52
		.30,.10	0.01, 0.01	-0.83	-0.98	-1.14	-1.16
			0.20, 0.05	-1.17	-1.21	-1.41	-1.35
			0.20, 0.20	-0.86	-0.97	-1.14	-1.13
			0.60, 0.01	-0.87	-0.95	-1.20	-1.11
$J = 5, n_j = 50$	Low	.20,.20	0.01, 0.01	0.11	0.12	-0.06	-0.05
			0.20, 0.05	-0.53	-0.44	-0.60	-0.66
			0.20, 0.20	-1.80	-1.63	-1.72	-1.97
			0.60, 0.01	0.19	0.15	-0.06	-0.06
		.30,.10	0.01, 0.01	0.01	-0.30	-0.39	-0.43
			0.20, 0.05	-0.57	-0.85	-0.92	-1.03
			0.20, 0.20	-1.81	-2.03	-2.05	-2.29
			0.60, 0.01	0.07	-0.28	-0.39	-0.41
	High	.20,.20	0.01, 0.01	0.21	-0.30	-0.49	-0.34
			0.20, 0.05	-0.34	-0.75	-2.32	-0.96
			0.20, 0.20	-1.69	-1.77	-2.39	-2.04
			0.60, 0.01	0.22	-0.21	0.07	-0.28
		.30,.10	0.01, 0.01	0.47	-0.21	-0.54	-0.18
			0.20, 0.05	-0.12	-0.71	-1.33	-0.62
			0.20, 0.20	-0.91	-1.78	-2.99	-1.58
			0.60, 0.01	0.40	-0.16	-1.11	-0.18
$J = 15$, unbalanced	Low	.20,.20	0.01, 0.01	-1.35	-0.46	-0.32	-0.42
			0.20, 0.05	-1.89	-0.95	-0.85	-0.88
			0.20, 0.20	-3.18	-2.02	-2.10	-1.95
			0.60, 0.01	-1.33	-0.50	-0.36	-0.42
		.30,.10	0.01, 0.01	-0.91	-0.26	-0.34	-0.38
			0.20, 0.05	-1.33	-0.78	-0.89	-0.94
			0.20, 0.20	-2.54	-1.84	-2.06	-2.12
			0.60, 0.01	-0.82	-0.31	-0.39	-0.40
	High	.20,.20	0.01, 0.01	-1.44	-0.81	-0.75	-0.83
			0.20, 0.05	-1.94	-1.26	-1.38	-1.32
			0.20, 0.20	-3.11	-2.26	-2.57	-2.17
			0.60, 0.01	-1.48	-0.91	-0.77	-0.88
		.30,.10	0.01, 0.01	-1.09	-0.45	-0.67	-0.65
			0.20, 0.05	-1.62	-0.91	-1.20	-1.13
			0.20, 0.20	-3.23	-1.98	-2.45	-2.36
			0.60, 0.01	-1.06	-0.51	-0.65	-0.63

Table A13: Percentage bias for treatment effect on Y_2 for scenarios corresponding to missing mechanism depending on both individual and cluster-level covariates

Design	η	Prob. nonresponse	ICC	CCA	SMI	FMI	MMI
$J = 25$, $n_j = 10$	Low	.20,.20	0.01, 0.01	-1.59	-2.67	-2.15	-2.29
			0.20, 0.05	-1.84	-2.93	-2.37	-2.54
			0.20, 0.20	-2.29	-3.48	-2.82	-3.00
			0.60, 0.01	-1.58	-2.68	-2.17	-2.29
		.30,.10	0.01, 0.01	-0.66	-1.71	-1.66	-1.59
			0.20, 0.05	-1.12	-1.95	-1.86	-1.83
			0.20, 0.20	-1.61	-2.46	-2.31	-2.30
			0.60, 0.01	-0.72	-1.70	-1.64	-1.57
	High	.20,.20	0.01, 0.01	-0.35	-2.40	-2.31	-2.49
			0.20, 0.05	-0.65	-2.72	-2.47	-2.84
			0.20, 0.20	-1.17	-3.35	-2.82	-3.54
			0.60, 0.01	-0.31	-2.43	-2.35	-2.49
		.30,.10	0.01, 0.01	-0.22	-2.29	-2.57	-2.47
			0.20, 0.05	-0.54	-2.49	-2.81	-2.68
			0.20, 0.20	-1.04	-2.92	-3.29	-3.20
			0.60, 0.01	-0.23	-2.30	-2.61	-2.44
$J = 5$, $n_j = 50$	Low	.20,.20	0.01, 0.01	-0.21	0.06	-0.01	-0.03
			0.20, 0.05	-0.87	-0.49	-0.55	-0.63
			0.20, 0.20	-2.14	-1.67	-1.67	-1.92
			0.60, 0.01	-0.24	0.09	-0.01	0.02
		.30,.10	0.01, 0.01	-0.39	-0.25	-0.35	-0.48
			0.20, 0.05	-1.04	-0.81	-0.88	-1.06
			0.20, 0.20	-2.47	-2.00	-2.01	-2.28
			0.60, 0.01	-0.46	-0.26	-0.35	-0.43
	High	.20,.20	0.01, 0.01	-0.48	-0.16	-1.23	-0.42
			0.20, 0.05	-1.14	-0.56	-1.69	-0.96
			0.20, 0.20	-2.22	-1.49	-2.81	-2.03
			0.60, 0.01	-0.65	-0.10	-0.67	-0.52
		.30,.10	0.01, 0.01	-0.12	-0.07	-0.42	-0.52
			0.20, 0.05	-1.02	-0.50	-0.95	-1.01
			0.20, 0.20	-2.33	-1.41	-2.02	-2.20
			0.60, 0.01	-0.39	-0.07	-0.44	-0.45
$J = 15$, unbalanced	Low	.20,.20	0.01, 0.01	-0.96	-0.16	0.37	-0.06
			0.20, 0.05	-1.45	-0.70	-0.21	-0.53
			0.20, 0.20	-2.87	-1.83	-1.45	-1.59
			0.60, 0.01	-0.92	-0.18	0.33	-0.05
		.30,.10	0.01, 0.01	-0.85	-0.15	-0.18	-0.08
			0.20, 0.05	-1.27	-0.74	-0.79	-0.67
			0.20, 0.20	-2.75	-1.88	-1.96	-1.84
			0.60, 0.01	-0.91	-0.19	-0.25	-0.07
	High	.20,.20	0.01, 0.01	-0.97	-0.39	-1.04	-0.58
			0.20, 0.05	-1.51	-0.92	-1.66	-1.13
			0.20, 0.20	-2.82	-2.17	-3.00	-2.20
			0.60, 0.01	-0.91	-0.19	-0.25	-0.07
		.30,.10	0.01, 0.01	-0.09	-0.39	-0.06	-0.39
			0.20, 0.05	-0.43	-0.95	-0.66	-1.03
			0.20, 0.20	-1.67	-2.14	-1.98	-2.26
			0.60, 0.01	-0.18	-0.50	-0.06	-0.43

Table A14: Percentage bias for treatment effect on Y_2 for scenarios corresponding to missing mechanism differential by treatment

Design	η	Prob. nonresponse	ICC	CCA	SMI	FMI	MMI
$J = 25$, $n_j = 10$	Low	.20,.20	0.01, 0.01	-26.5	-2.6	-1.5	-2.3
			0.20, 0.05	-29.9	-2.9	-1.8	-2.5
			0.20, 0.20	-35.8	-3.5	-2.5	-3.0
			0.60, 0.01	-26.6	-2.7	-1.4	-2.3
		.30,.10	0.01, 0.01	-24.4	-1.7	-1.1	-1.8
			0.20, 0.05	-27.8	-1.9	-1.4	-2.0
			0.20, 0.20	-33.4	-2.4	-1.9	-2.5
			0.60, 0.01	-24.6	-1.7	-1.1	-1.8
	High	.20,.20	0.01, 0.01	-30.5	-2.1	-1.6	-2.7
			0.20, 0.05	-34.6	-2.4	-2.0	-2.9
			0.20, 0.20	-41.4	-3.0	-3.0	-3.4
			0.60, 0.01	-30.7	-2.1	-1.6	-2.6
		.30,.10	0.01, 0.01	-31.6	-2.2	-1.6	-2.9
			0.20, 0.05	-35.9	-2.5	-2.0	-3.2
			0.20, 0.20	-43.4	-3.1	-2.9	-3.7
			0.60, 0.01	-31.8	-2.2	-1.5	-2.9
$J = 5$, $n_j = 50$	Low	.20,.20	0.01, 0.01	-27.1	-0.6	-0.5	-0.6
			0.20, 0.05	-30.6	-1.2	-1.0	-1.2
			0.20, 0.20	-34.6	-2.6	-2.2	-2.4
			0.60, 0.01	-27.4	-0.7	-0.5	-0.7
		.30,.10	0.01, 0.01	-27.0	-0.6	-0.5	-0.4
			0.20, 0.05	-30.2	-1.2	-1.0	-0.9
			0.20, 0.20	-34.1	-2.6	-2.2	-2.1
			0.60, 0.01	-27.3	-0.6	-0.5	-0.4
	High	.20,.20	0.01, 0.01	-31.4	-0.6	-0.6	-0.4
			0.20, 0.05	-35.3	-1.3	-1.1	-0.9
			0.20, 0.20	-40.0	-2.6	-2.3	-2.1
			0.60, 0.01	-31.7	-0.7	-0.6	-0.4
		.30,.10	0.01, 0.01	-33.8	-0.6	-0.6	-0.6
			0.20, 0.05	-37.9	-1.2	-1.2	-1.2
			0.20, 0.20	-43.3	-2.5	-2.4	-2.3
			0.60, 0.01	-34.1	-0.6	-0.6	-0.6
$J = 15$, unbalanced	Low	.20,.20	0.01, 0.01	-26.1	-0.2	-0.1	-0.5
			0.20, 0.05	-29.7	-0.7	-0.8	-1.2
			0.20, 0.20	-35.6	-1.9	-2.2	-2.5
			0.60, 0.01	-26.3	-0.2	-0.2	-0.6
		.30,.10	0.01, 0.01	-25.3	0.0	0.2	0.0
			0.20, 0.05	-28.7	-0.6	-0.4	-0.6
			0.20, 0.20	-34.4	-1.9	-1.6	-1.8
			0.60, 0.01	-25.4	-0.1	0.2	0.0
	High	.20,.20	0.01, 0.01	-30.2	-0.4	-0.6	-0.5
			0.20, 0.05	-34.6	-0.9	-1.3	-1.0
			0.20, 0.20	-41.7	-2.2	-2.8	-2.4
			0.60, 0.01	-30.5	-0.4	-0.6	-0.5
		.30,.10	0.01, 0.01	-31.7	-0.3	-0.2	-0.6
			0.20, 0.05	-36.3	-0.8	-0.9	-1.1
			0.20, 0.20	-43.8	-2.1	-2.4	-2.4
			0.60, 0.01	-32.0	-0.3	-0.3	-0.5

Table A15: Coverage rate and average width (AW) of the 95% CI for treatment effect on Y_2 after each of the MI strategies, when the missing mechanism depends only on individual level covariate

Design	η	Prob. Miss	ICC	CCA		SMI		FMI		MMI	
				CR	AW	CR	AW	CR	AW	CR	AW
$J = 25$, $n_j = 10$	Low	.20,.20	0.01, 0.01	94.4	9.4	94.8	8.5	97.3	10.1	95.8	8.7
			0.20, 0.05	94.0	10.3	94.1	9.2	96.5	11.0	94.5	9.5
			0.20, 0.20	93.8	14.3	90.4	12.1	95.5	14.8	93.0	13.5
			0.60, 0.01	94.7	9.4	95.1	8.6	97.2	10.0	95.8	8.7
		.30,.10	0.01, 0.01	95.5	9.5	95.2	8.0	96.7	8.8	95.7	8.1
			0.20, 0.05	94.8	10.4	93.4	8.9	95.9	9.8	94.1	9.0
			0.20, 0.20	93.6	14.3	91.8	12.4	94.5	13.9	93.8	13.3
			0.60, 0.01	95.7	9.5	95.6	8.1	96.5	8.7	95.6	8.1
	High	.20,.20	0.01, 0.01	96.2	9.4	94.7	9.3	96.9	11.3	94.3	9.4
			0.20, 0.05	95.6	10.3	92.2	9.8	96.3	12.2	93.5	10.1
			0.20, 0.20	93.9	14.3	89.5	12.4	95.6	15.7	92.8	13.9
			0.60, 0.01	96.2	9.4	94.8	9.3	96.7	11.2	94.0	9.4
		.30,.10	0.01, 0.01	95.4	9.4	94.7	8.5	97.3	9.8	94.7	8.7
			0.20, 0.05	94.1	10.3	93.2	9.2	97.1	10.7	94.2	9.5
			0.20, 0.20	93.3	14.3	90.7	12.4	95.3	14.6	93.3	13.6
			0.60, 0.01	95.5	9.5	94.9	8.6	97.4	9.7	94.5	8.7
$J = 5$, $n_j = 50$	Low	.20,.20	0.01, 0.01	94.9	10.5	95.1	9.4	97.1	10.8	95.7	9.8
			0.20, 0.05	91.8	13.9	89.4	11.8	94.1	14.3	92.2	13.2
			0.20, 0.20	89.0	25.5	83.3	20.4	92.0	25.8	90.3	25.0
			0.60, 0.01	95.5	10.6	95.4	9.5	97.0	10.6	95.6	9.8
		.30,.10	0.01, 0.01	94.8	10.6	94.2	9.0	95.9	9.8	94.8	9.3
			0.20, 0.05	91.3	14.0	89.8	12.2	93.3	13.5	91.8	13.0
			0.20, 0.20	90.8	25.4	87.1	22.3	91.2	25.4	90.5	25.0
			0.60, 0.01	94.2	21.2	93.9	18.3	95.6	19.5	94.9	18.6
	High	.20,.20	0.01, 0.01	94.5	10.5	94.6	10.0	97.5	11.8	95.9	10.5
			0.20, 0.05	91.0	13.9	87.5	12.0	94.4	15.1	91.6	13.6
			0.20, 0.20	90.3	25.5	80.6	19.4	92.0	26.3	89.4	25.2
			0.60, 0.01	94.4	10.6	94.6	10.1	97.2	11.6	96.3	10.6
		.30,.10	0.01, 0.01	94.0	10.6	94.0	9.4	96.5	10.6	95.0	9.8
			0.20, 0.05	91.3	14.0	89.6	12.0	93.7	14.1	91.8	13.2
			0.20, 0.20	90.4	25.5	85.0	20.9	91.6	25.7	90.9	25.0
			0.60, 0.01	94.2	10.6	94.7	9.5	96.4	10.4	95.8	9.8
$J = 15$, unbalanced	Low	.20,.20	0.01, 0.01	95.0	9.0	92.7	8.1	96.8	9.7	93.6	8.3
			0.20, 0.05	93.3	10.7	90.9	9.3	96.1	11.3	91.9	9.8
			0.20, 0.20	93.3	10.7	90.9	9.3	96.1	11.3	91.9	9.8
			0.60, 0.01	94.8	9.0	93.4	8.2	96.4	9.5	93.4	8.3
		.30,.10	0.01, 0.01	94.2	9.0	94.4	7.7	96.0	8.5	93.9	7.8
			0.20, 0.05	91.8	10.6	91.5	9.3	94.2	10.3	92.7	9.6
			0.20, 0.20	92.7	16.7	90.3	14.6	94.5	16.6	93.5	16.0
			0.60, 0.01	94.4	9.1	94.3	7.8	95.6	8.4	94.3	7.8
	High	.20,.20	0.01, 0.01	93.6	9.0	94.5	8.7	97.5	10.8	94.9	9.0
			0.20, 0.05	92.9	10.6	91.4	9.8	97.3	12.3	93.1	10.4
			0.20, 0.20	93.2	16.8	86.5	13.6	95.9	18.0	92.7	16.4
			0.60, 0.01	94.5	9.0	94.6	8.8	97.3	10.6	94.9	9.0
		.30,.10	0.01, 0.01	94.4	9.0	94.3	8.1	96.3	9.3	94.6	8.3
			0.20, 0.05	93.3	10.7	92.2	9.4	96.0	11.1	93.2	9.9
			0.20, 0.20	93.9	16.8	89.2	14.1	94.8	17.1	93.3	16.2
			0.60, 0.01	94.3	9.1	94.3	8.2	96.1	9.2	94.7	8.3

Table A16: Coverage rate and average width (AW) of the 95 CI for treatment effect on Y_2 after each of the MI strategies, when the missing mechanism depends on cluster-level covariate.

Design	η	Prob. Miss	ICC	CCA		SMI		FMI		MMI	
				CR	AW	CR	AW	CR	AW	CR	AW
$J = 25$, $n_j = 10$	Low	.20,.20	0.01, 0.01	95.6	9.7	95.3	9.1	99.2	11.8	95.2	9.2
			0.20, 0.05	95.1	10.7	94.0	10.0	98.6	12.8	94.8	10.2
			0.20, 0.20	94.3	14.9	92.2	13.4	97.2	16.4	93.7	14.2
			0.60, 0.01	94.0	9.6	95.4	8.8	98.5	11.9	95.6	9.1
		.30,.10	0.01, 0.01	95.7	9.7	95.6	8.4	97.7	9.7	95.1	8.4
			0.20, 0.05	94.7	10.8	93.9	9.4	96.9	10.8	94.2	9.5
			0.20, 0.20	93.8	14.9	92.0	13.2	95.6	14.9	93.5	13.8
			0.60, 0.01	95.7	9.8	96.1	8.4	97.6	9.7	95.8	8.5
	High	.20,.20	0.01, 0.01	96.6	9.9	95.6	10.2	99.5	14.8	96.5	10.2
			0.20, 0.05	95.4	11.0	95.8	11.0	99.2	15.6	95.6	11.2
			0.20, 0.20	94.9	15.2	93.4	14.2	98.1	18.7	94.9	15.1
			0.60, 0.01	97.0	10.0	95.9	10.3	99.5	14.9	96.6	10.3
		.30,.10	0.01, 0.01	95.3	10.0	95.6	9.1	98.7	12.3	95.4	9.2
			0.20, 0.05	94.2	11.0	94.2	10.0	98.5	13.2	94.6	10.3
			0.20, 0.20	95.9	10.1	95.9	9.1	98.8	12.3	95.9	9.3
			0.60, 0.01	95.9	10.0	95.9	9.2	98.7	12.3	95.8	9.3
$J = 5$, $n_j = 50$	Low	.20,.20	0.01, 0.01	94.5	10.9	95.5	10.5	97.4	12.1	95.6	10.9
			0.20, 0.05	91.4	14.5	91.5	13.7	94.5	15.7	93.1	14.6
			0.20, 0.20	90.4	26.3	88.5	23.8	92.4	27.3	91.3	26.4
			0.60, 0.01	94.5	11.1	95.7	10.6	97.2	11.9	96.2	11.0
		.30,.10	0.01, 0.01	94.3	10.9	94.6	9.7	96.3	10.5	94.5	9.9
			0.20, 0.05	91.0	14.6	90.9	13.3	92.8	14.4	92.2	13.9
			0.20, 0.20	90.1	26.1	89.1	24.5	91.4	26.5	90.8	26.1
			0.60, 0.01	94.7	11.1	95.2	9.7	96.1	10.4	94.9	10.0
	High	.20,.20	0.01, 0.01	93.2	11.4	95.4	12.4	97.8	17.6	96.4	13.0
			0.20, 0.05	90.3	14.9	91.0	15.2	95.6	20.5	92.7	16.6
			0.20, 0.20	90.2	26.5	87.5	24.8	93.1	30.9	91.0	28.0
			0.60, 0.01	93.5	11.6	95.7	12.4	97.8	18.3	97.2	13.3
		.30,.10	0.01, 0.01	94.6	11.3	94.8	10.9	98.0	13.5	95.6	11.4
			0.20, 0.05	90.2	15.0	91.2	14.1	94.5	17.5	92.9	15.0
			0.20, 0.20	89.0	26.4	89.3	24.7	92.0	28.5	90.7	26.9
			0.60, 0.01	95.2	11.6	95.1	10.9	97.8	14.7	95.3	11.5
$J = 15$, unbalanced	Low	.20,.20	0.01, 0.01	94.1	9.2	95.1	8.8	98.4	11.2	95.3	8.9
			0.20, 0.05	93.0	11.1	93.2	10.3	97.7	12.9	94.3	10.7
			0.20, 0.20	93.9	17.4	90.7	15.5	96.3	18.8	93.4	17.0
			0.60, 0.01	94.7	9.3	95.3	8.8	98.3	11.1	95.6	8.9
		.30,.10	0.01, 0.01	94.4	9.3	95.2	8.1	97.0	9.3	95.1	8.2
			0.20, 0.05	93.0	11.2	92.4	9.9	96.0	11.3	92.8	10.1
			0.20, 0.20	92.8	17.4	92.5	15.7	94.6	17.6	93.4	16.7
			0.60, 0.01	94.3	9.4	95.7	8.1	96.8	9.2	95.5	8.2
	High	.20,.20	0.01, 0.01	93.6	9.5	94.5	9.9	98.7	14.9	95.1	10.1
			0.20, 0.05	92.3	11.5	93.2	11.3	98.1	16.3	93.8	11.7
			0.20, 0.20	92.7	17.8	90.1	16.2	96.7	21.2	92.7	17.8
			0.60, 0.01	94.4	9.7	95.0	9.9	98.5	15.0	95.2	10.1
		.30,.10	0.01, 0.01	93.8	9.6	95.1	8.8	98.5	12.0	95.7	9.0
			0.20, 0.05	92.2	11.4	93.1	10.4	98.0	13.6	94.4	10.9
			0.20, 0.20	92.4	17.8	90.5	16.0	96.0	19.2	93.4	17.3
			0.60, 0.01	94.0	9.6	95.4	8.8	98.5	11.9	95.6	9.1

Table A17: Coverage rate and average width (AW) of the 95 CI for treatment effect on Y_2 after each of the MI strategies, when the missing mechanism depends on both individual and cluster-level covariates

Design	η	Prob. Miss	ICC	CCA		SMI		FMI		MMI	
				CR	AW	CR	AW	CR	AW	CR	AW
$J = 25$, $n_j = 10$	Low	.20,.20	0.01, 0.01	95.2	9.6	95.4	9.0	98.8	11.2	95.3	9.1
			0.20, 0.05	93.7	10.3	94.5	9.9	98.3	12.1	94.7	10.1
			0.20, 0.20	94.8	14.0	91.6	13.3	96.2	15.9	93.8	14.2
			0.60, 0.01	95.7	9.6	95.7	9.1	98.7	11.1	95.4	9.2
		.30,.10	0.01, 0.01	95.6	9.6	95.3	8.3	97.1	9.4	95.5	8.4
			0.20, 0.05	94.9	10.3	94.2	9.3	96.8	10.5	94.3	9.5
			0.20, 0.20	93.9	14.1	91.9	13.2	95.5	14.7	94.2	13.9
			0.60, 0.01	95.7	9.7	95.5	8.4	97.4	9.4	95.6	8.4
	High	.20,.20	0.01, 0.01	95.5	9.8	95.3	10.4	99.0	14.0	95.3	10.5
			0.20, 0.05	95.1	10.4	94.4	11.2	98.6	14.7	94.3	11.4
			0.20, 0.20	95.0	13.9	92.9	14.3	97.5	17.9	94.2	15.3
			0.60, 0.01	96.3	9.9	95.9	10.5	98.8	14.0	95.4	10.5
		.30,.10	0.01, 0.01	96.1	9.9	95.3	9.4	98.5	12.0	95.3	9.5
			0.20, 0.05	95.6	10.5	94.2	10.2	97.9	12.9	94.1	10.5
			0.20, 0.20	94.5	14.0	91.1	13.7	96.5	16.5	94.0	14.6
			0.60, 0.01	96.1	10.0	95.6	9.5	98.5	11.9	95.2	9.5
$J = 5$, $n_j = 50$	Low	.20,.20	0.01, 0.01	95.2	10.7	95.5	10.4	97.3	11.7	96.0	10.7
			0.20, 0.05	92.3	13.3	90.3	13.5	94.2	15.4	92.2	14.4
			0.20, 0.20	90.7	24.0	87.7	23.7	91.6	27.2	90.4	26.4
			0.60, 0.01	95.2	10.9	95.6	10.5	96.9	11.6	96.5	10.8
		.30,.10	0.01, 0.01	94.6	10.7	95.0	9.7	96.2	10.3	95.2	9.8
			0.20, 0.05	90.3	13.4	90.6	13.2	92.1	14.3	91.7	13.8
			0.20, 0.20	89.4	24.1	88.7	24.4	91.0	26.5	90.7	26.1
			0.60, 0.01	95.2	10.9	94.7	9.7	96.1	10.2	95.1	9.9
	High	.20,.20	0.01, 0.01	93.1	12.0	95.7	12.2	97.7	15.6	96.9	12.6
			0.20, 0.05	91.8	13.8	92.3	14.9	95.3	18.7	94.0	16.0
			0.20, 0.20	89.9	23.2	87.7	24.2	92.2	28.8	91.4	27.3
			0.60, 0.01	93.0	12.0	95.9	12.2	97.9	14.4	96.9	12.8
		.30,.10	0.01, 0.01	94.0	12.0	96.1	10.9	97.4	12.3	96.2	11.2
			0.20, 0.05	92.4	13.8	91.2	14.0	94.1	15.8	92.3	14.8
			0.20, 0.20	89.8	23.2	88.6	24.1	91.9	27.3	90.7	26.7
			0.60, 0.01	94.6	11.9	96.3	10.9	97.1	12.2	96.6	11.3
$J = 15$, unbalanced	Low	.20,.20	0.01, 0.01	94.2	9.1	94.6	8.6	97.6	10.6	95.1	8.8
			0.20, 0.05	93.0	10.4	92.2	10.2	97.2	12.3	94.2	10.5
			0.20, 0.20	94.7	16.3	89.9	15.4	96.1	18.4	93.5	16.9
			0.60, 0.01	94.5	9.2	94.8	8.8	97.4	10.5	95.1	8.8
		.30,.10	0.01, 0.01	94.4	9.1	93.9	8.0	96.5	9.0	94.3	8.1
			0.20, 0.05	93.1	10.4	92.3	9.8	95.3	11.0	92.9	10.1
			0.20, 0.20	93.1	16.3	91.1	15.7	93.8	17.4	93.6	16.7
			0.60, 0.01	94.7	9.2	94.3	8.1	96.2	8.9	94.4	8.2
	High	.20,.20	0.01, 0.01	93.9	9.6	95.0	10.0	98.1	13.5	95.4	10.2
			0.20, 0.05	92.2	10.7	92.4	11.3	97.9	14.9	93.6	11.8
			0.20, 0.20	92.5	16.0	90.5	16.1	96.5	20.2	94.0	17.8
			0.60, 0.01	94.7	9.2	94.3	8.1	96.2	8.9	94.4	8.2
		.30,.10	0.01, 0.01	94.1	9.7	94.6	9.0	97.8	11.4	95.3	9.2
			0.20, 0.05	92.6	10.7	92.3	10.5	96.5	13.0	93.4	10.9
			0.20, 0.20	93.3	16.0	90.4	15.8	95.4	18.8	93.7	17.3
			0.60, 0.01	94.4	9.7	94.4	9.1	97.5	11.3	95.1	9.2

Table A18: Coverage rate and average width (AW) of the 95% CI for treatment effect on Y_2 after each of the MI strategies, when the missing mechanism is differential by treatment arm.

Design	η	Prob. Miss	ICC	CCA		SMI		FMI		MMI	
				CR	AW	CR	AW	CR	AW	CR	AW
$J = 25$, $n_j = 10$	Low	.20,.20	0.01, 0.01	81.2	18.6	95.5	17.9	98.8	22.3	94.9	17.5
			0.20, 0.05	84.7	27.6	92.6	26.2	96.5	31.2	93.1	27.4
			0.20, 0.20	83.9	27.7	92.8	26.3	96.3	31.2	93.1	27.2
			0.60, 0.01	91.3	56.0	90.7	51.4	95.1	58.7	94.4	56.9
		.30,.10	0.01, 0.01	82.2	18.7	95.5	19.4	99.7	26.1	94.9	18.9
			0.20, 0.05	85.2	27.7	92.5	26.9	97.5	34.0	93.0	28.2
			0.20, 0.20	84.6	27.7	92.6	27.0	97.5	34.0	92.4	27.9
			0.60, 0.01	91.7	56.0	90.7	50.8	95.2	60.1	93.5	57.5
	High	.20,.20	0.01, 0.01	75.1	17.4	95.6	17.3	98.7	21.4	95.6	17.2
			0.20, 0.05	81.2	26.7	91.8	26.2	96.8	30.6	93.8	27.4
			0.20, 0.20	81.2	26.6	91.9	26.2	96.8	30.6	93.7	27.2
			0.60, 0.01	89.7	55.2	91.0	52.3	94.6	58.4	94.4	56.9
		.30,.10	0.01, 0.01	73.7	17.8	96.2	18.5	99.1	23.4	95.8	18.1
			0.20, 0.05	78.4	26.8	92.2	26.8	96.8	32.0	93.2	28.0
			0.20, 0.20	78.6	26.8	92.3	26.8	96.8	32.1	93.6	27.8
			0.60, 0.01	89.3	55.3	90.4	52.2	95.0	59.1	94.4	57.4
$J = 5$, $n_j = 50$	Low	.20,.20	0.01, 0.01	82.0	20.3	94.8	20.2	96.5	22.6	95.6	20.2
			0.20, 0.05	87.4	48.1	87.4	47.0	92.4	53.1	91.0	51.1
			0.20, 0.20	87.2	48.4	87.8	47.0	92.6	53.1	90.9	51.1
			0.60, 0.01	89.9	116.1	87.5	107.3	91.1	121.5	90.9	120.2
		.30,.10	0.01, 0.01	83.2	20.3	94.8	21.8	97.2	25.4	95.3	21.7
			0.20, 0.05	86.6	47.9	87.2	46.3	92.1	54.3	90.3	51.2
			0.20, 0.20	87.7	48.2	87.2	46.4	92.4	54.1	90.2	51.0
			0.60, 0.01	89.0	115.7	85.3	103.7	91.0	121.5	90.7	120.0
	High	.20,.20	0.01, 0.01	75.6	19.4	94.8	19.8	96.1	21.9	94.1	20.0
			0.20, 0.05	85.4	47.3	88.9	47.9	92.0	52.7	91.4	51.3
			0.20, 0.20	85.3	47.5	88.9	47.9	92.1	52.6	91.4	51.3
			0.60, 0.01	89.7	115.0	87.9	110.2	91.2	121.0	91.0	120.2
		.30,.10	0.01, 0.01	74.3	20.0	94.6	20.9	97.1	27.0	95.1	21.0
			0.20, 0.05	85.1	47.6	87.6	47.7	92.8	54.3	90.1	51.7
			0.20, 0.20	84.9	47.8	87.5	47.7	92.4	53.8	90.2	51.6
			0.60, 0.01	89.1	115.0	86.9	108.4	91.0	123.7	90.8	120.9
$J = 15$, unbalanced	Low	.20,.20	0.01, 0.01	81.0	17.7	93.9	17.0	97.4	21.0	93.2	16.9
			0.20, 0.05	85.9	32.1	89.9	30.3	95.7	36.1	93.0	32.9
			0.20, 0.20	85.9	32.1	89.7	30.3	96.0	35.9	93.1	32.7
			0.60, 0.01	91.2	70.3	89.6	63.7	94.3	74.0	93.5	72.4
		.30,.10	0.01, 0.01	82.6	17.8	93.8	18.4	98.5	24.6	93.9	18.3
			0.20, 0.05	85.7	32.1	88.0	30.4	96.4	38.3	92.2	33.4
			0.20, 0.20	85.5	32.1	88.2	30.4	96.2	38.1	91.5	33.1
			0.60, 0.01	91.9	70.5	87.4	62.1	94.3	75.1	94.0	72.9
	High	.20,.20	0.01, 0.01	74.9	16.6	93.5	16.7	97.6	20.4	93.6	16.8
			0.20, 0.05	83.0	31.1	90.9	30.8	96.0	35.6	92.9	33.0
			0.20, 0.20	83.0	31.2	90.6	30.7	95.9	35.5	92.8	32.8
			0.60, 0.01	91.0	69.5	89.7	65.5	94.6	73.6	93.8	72.4
		.30,.10	0.01, 0.01	73.4	17.1	94.3	17.5	97.4	22.9	93.8	17.7
			0.20, 0.05	82.5	31.4	90.7	30.8	96.0	37.3	93.5	33.4
			0.20, 0.20	82.1	31.4	91.0	30.8	96.2	36.9	93.0	33.1
			0.60, 0.01	90.5	69.7	89.7	64.5	94.0	74.4	93.8	72.8

Table A19: RMSE for treatment estimate on Y_2 after each of the MI methods under comparison, for scenarios corresponding to missing data mechanisms depending on individual-level covariate

Design	η	Prob. nonresponse	ICC	CCA	SMI	FMI	MMI
$J = 25$, $n_j = 10$	Low	.20,.20	0.01, 0.01	2.33	2.13	2.16	2.14
			0.20, 0.05	2.66	2.47	2.49	2.48
			0.20, 0.20	3.76	3.62	3.61	3.61
			0.60, 0.01	2.34	2.13	2.16	2.15
		.30,.10	0.01, 0.01	2.33	2.00	2.01	1.99
			0.20, 0.05	2.65	2.35	2.35	2.34
			0.20, 0.20	3.74	3.52	3.49	3.49
			0.60, 0.01	2.33	2.00	2.01	1.99
	High	.20,.20	0.01, 0.01	2.32	2.40	2.46	2.38
			0.20, 0.05	2.64	2.72	2.76	2.70
			0.20, 0.20	3.74	3.85	3.82	3.80
			0.60, 0.01	2.32	2.40	2.46	2.39
		.30,.10	0.01, 0.01	2.35	2.19	2.20	2.17
			0.20, 0.05	2.66	2.52	2.51	2.49
			0.20, 0.20	3.76	3.66	3.62	3.60
			0.60, 0.01	2.35	2.19	2.20	2.17
$J = 5$, $n_j = 50$	Low	.20,.20	0.01, 0.01	2.61	2.37	2.38	2.39
			0.20, 0.05	3.77	3.58	3.58	3.60
			0.20, 0.20	6.98	6.85	6.83	6.86
			0.60, 0.01	2.61	2.37	2.38	2.39
		.30,.10	0.01, 0.01	2.64	2.29	2.29	2.30
			0.20, 0.05	3.81	3.53	3.52	3.54
			0.20, 0.20	7.02	6.82	6.81	6.82
			0.60, 0.01	5.26	4.56	4.55	4.56
	High	.20,.20	0.01, 0.01	2.66	2.57	2.60	2.57
			0.20, 0.05	3.81	3.71	3.74	3.73
			0.20, 0.20	7.01	6.92	6.94	6.94
			0.60, 0.01	2.66	2.56	2.60	2.57
		.30,.10	0.01, 0.01	2.68	2.43	2.42	2.43
			0.20, 0.05	3.81	3.63	3.62	3.63
			0.20, 0.20	7.00	6.89	6.88	6.88
			0.60, 0.01	2.67	2.43	2.42	2.43
$J = 15$, unbalanced	Low	.20,.20	0.01, 0.01	2.31	2.15	2.20	2.16
			0.20, 0.05	2.83	2.70	2.73	2.69
			0.20, 0.20	2.83	2.70	2.73	2.69
			0.60, 0.01	2.30	2.14	2.19	2.15
		.30,.10	0.01, 0.01	2.36	2.01	2.05	2.02
			0.20, 0.05	2.88	2.59	2.62	2.60
			0.20, 0.20	4.42	4.25	4.26	4.24
			0.60, 0.01	2.36	2.01	2.05	2.02
	High	.20,.20	0.01, 0.01	2.29	2.24	2.30	2.24
			0.20, 0.05	2.83	2.76	2.79	2.76
			0.20, 0.20	4.41	4.36	4.32	4.33
			0.60, 0.01	2.30	2.24	2.30	2.25
		.30,.10	0.01, 0.01	2.28	2.11	2.13	2.11
			0.20, 0.05	2.80	2.67	2.67	2.66
			0.20, 0.20	4.37	4.31	4.28	4.27
			0.60, 0.01	2.29	2.11	2.13	2.11

Table A20: RMSE for treatment estimate on Y_2 after each of the MI methods under comparison, for scenarios corresponding to missing data mechanisms depending on cluster-level covariates

Design	η	Prob. nonresponse	ICC	CCA	SMI	FMI	MMI
$J = 25$, $n_j = 10$	Low	.20,.20	0.01, 0.01	2.45	2.25	2.34	2.25
			0.20, 0.05	2.78	2.60	2.68	2.60
			0.20, 0.20	3.94	3.80	3.84	3.79
			0.60, 0.01	2.46	2.20	2.38	2.18
		.30,.10	0.01, 0.01	2.46	2.09	2.12	2.09
			0.20, 0.05	2.80	2.46	2.49	2.46
			0.20, 0.20	3.95	3.70	3.71	3.70
			0.60, 0.01	2.46	2.08	2.12	2.09
	High	.20,.20	0.01, 0.01	2.47	2.44	2.71	2.45
			0.20, 0.05	2.79	2.75	2.98	2.74
			0.20, 0.20	3.96	3.93	4.04	3.86
			0.60, 0.01	2.47	2.45	2.70	2.46
		.30,.10	0.01, 0.01	2.52	2.22	2.44	2.22
			0.20, 0.05	2.85	2.58	2.77	2.57
			0.20, 0.20	2.52	2.22	2.44	2.22
			0.60, 0.01	2.53	2.22	2.45	2.23
$J = 5$, $n_j = 50$	Low	.20,.20	0.01, 0.01	2.75	2.54	2.56	2.57
			0.20, 0.05	3.92	3.74	3.76	3.79
			0.20, 0.20	7.15	7.08	7.08	7.13
			0.60, 0.01	2.76	2.54	2.56	2.58
		.30,.10	0.01, 0.01	2.73	2.42	2.41	2.43
			0.20, 0.05	3.92	3.68	3.68	3.70
			0.20, 0.20	7.18	7.06	7.06	7.09
			0.60, 0.01	2.74	2.42	2.41	2.43
	High	.20,.20	0.01, 0.01	2.85	2.94	3.33	2.91
			0.20, 0.05	4.05	4.10	4.53	4.06
			0.20, 0.20	7.22	7.45	7.53	7.29
			0.60, 0.01	2.87	2.94	3.54	2.96
		.30,.10	0.01, 0.01	2.84	2.58	2.73	2.57
			0.20, 0.05	4.05	3.82	3.95	3.79
			0.20, 0.20	7.30	7.20	7.25	7.14
			0.60, 0.01	2.87	2.58	2.87	2.60
$J = 15$, unbalanced	Low	.20,.20	0.01, 0.01	2.36	2.19	2.28	2.19
			0.20, 0.05	2.92	2.75	2.83	2.75
			0.20, 0.20	4.58	4.47	4.49	4.45
			0.60, 0.01	2.36	2.19	2.27	2.18
		.30,.10	0.01, 0.01	2.41	2.05	2.09	2.06
			0.20, 0.05	2.96	2.66	2.69	2.66
			0.20, 0.20	4.57	4.40	4.41	4.38
			0.60, 0.01	2.41	2.05	2.09	2.06
	High	.20,.20	0.01, 0.01	2.49	2.43	2.78	2.44
			0.20, 0.05	3.09	3.02	3.24	3.00
			0.20, 0.20	4.80	4.80	4.80	4.69
			0.60, 0.01	2.48	2.44	2.78	2.47
		.30,.10	0.01, 0.01	2.47	2.19	2.38	2.18
			0.20, 0.05	3.06	2.80	2.94	2.78
			0.20, 0.20	4.72	4.59	4.62	4.53
			0.60, 0.01	2.46	2.20	2.38	2.18

Table A21: RMSE for treatment estimate on Y_2 after each of the MI methods under comparison, for scenarios corresponding to missing data mechanisms depending on both individual and cluster-level covariates

Design	η	Prob. nonresponse	ICC	CCA	SMI	FMI	MMI
$J = 25$, $n_j = 10$	Low	.20,.20	0.01, 0.01	2.39	2.25	2.29	2.24
			0.20, 0.05	2.65	2.59	2.64	2.60
			0.20, 0.20	3.70	3.80	3.81	3.80
			0.60, 0.01	2.39	2.24	2.29	2.25
		.30,.10	0.01, 0.01	2.34	2.07	2.07	2.05
			0.20, 0.05	2.61	2.44	2.44	2.43
			0.20, 0.20	3.68	3.69	3.67	3.67
			0.60, 0.01	2.34	2.07	2.07	2.05
	High	.20,.20	0.01, 0.01	2.42	2.54	2.68	2.55
			0.20, 0.05	2.63	2.87	2.96	2.88
			0.20, 0.20	3.62	4.07	4.04	4.03
			0.60, 0.01	2.43	2.54	2.68	2.56
		.30,.10	0.01, 0.01	2.41	2.33	2.46	2.34
			0.20, 0.05	2.61	2.68	2.78	2.68
			0.20, 0.20	3.58	3.89	3.92	3.83
			0.60, 0.01	2.42	2.33	2.45	2.35
$J = 5$, $n_j = 50$	Low	.20,.20	0.01, 0.01	2.66	2.50	2.52	2.52
			0.20, 0.05	3.59	3.72	3.73	3.75
			0.20, 0.20	6.62	7.07	7.06	7.10
			0.60, 0.01	2.66	2.51	2.52	2.53
		.30,.10	0.01, 0.01	2.64	2.40	2.40	2.39
			0.20, 0.05	3.60	3.68	3.68	3.67
			0.20, 0.20	6.67	7.07	7.07	7.07
			0.60, 0.01	2.66	2.40	2.40	2.38
	High	.20,.20	0.01, 0.01	2.99	2.88	3.41	2.81
			0.20, 0.05	3.74	4.04	4.32	3.97
			0.20, 0.20	6.55	7.35	7.41	7.25
			0.60, 0.01	3.05	2.88	2.95	2.85
		.30,.10	0.01, 0.01	2.96	2.59	2.63	2.59
			0.20, 0.05	3.67	3.80	3.82	3.80
			0.20, 0.20	6.49	7.15	7.13	7.13
			0.60, 0.01	3.00	2.59	2.63	2.59
$J = 15$, unbalanced	Low	.20,.20	0.01, 0.01	2.34	2.20	2.26	2.18
			0.20, 0.05	2.78	2.78	2.81	2.74
			0.20, 0.20	4.33	4.52	4.48	4.44
			0.60, 0.01	2.35	2.20	2.25	2.19
		.30,.10	0.01, 0.01	2.37	2.08	2.09	2.09
			0.20, 0.05	2.81	2.68	2.69	2.69
			0.20, 0.20	4.31	4.42	4.40	4.40
			0.60, 0.01	2.38	2.08	2.08	2.09
	High	.20,.20	0.01, 0.01	2.50	2.52	2.76	2.54
			0.20, 0.05	2.85	3.05	3.23	3.06
			0.20, 0.20	4.30	4.75	4.76	4.66
			0.60, 0.01	2.38	2.08	2.08	2.09
		.30,.10	0.01, 0.01	2.50	2.26	2.37	2.24
			0.20, 0.05	2.87	2.83	2.92	2.81
			0.20, 0.20	4.29	4.57	4.56	4.50
			0.60, 0.01	2.51	2.26	2.37	2.25

Table A22: RMSE for treatment estimate on Y_2 after each of the MI methods under comparison, for scenarios corresponding to missing data mechanisms differential by treatment arm.

Design	η	Prob. nonresponse	ICC	CCA	SMI	FMI	MMI
$J = 25$, $n_j = 10$	Low	.20,.20	0.01, 0.01	6.86	4.37	4.63	4.39
			0.20, 0.05	9.69	7.14	7.25	7.16
			0.20, 0.20	9.72	7.14	7.25	7.16
			0.60, 0.01	16.17	14.77	14.63	14.64
		.30,.10	0.01, 0.01	6.64	4.74	4.96	4.71
			0.20, 0.05	9.48	7.47	7.49	7.45
			0.20, 0.20	9.49	7.47	7.49	7.48
			0.60, 0.01	16.06	15.11	14.77	14.76
	High	.20,.20	0.01, 0.01	7.15	4.32	4.46	4.30
			0.20, 0.05	10.12	7.24	7.15	7.09
			0.20, 0.20	10.17	7.24	7.15	7.10
			0.60, 0.01	16.46	15.02	14.60	14.60
		.30,.10	0.01, 0.01	7.36	4.42	4.65	4.46
			0.20, 0.05	10.44	7.32	7.25	7.26
			0.20, 0.20	10.51	7.33	7.26	7.27
			0.60, 0.01	16.90	15.16	14.60	14.65
$J = 5$, $n_j = 50$	Low	.20,.20	0.01, 0.01	7.40	5.20	5.20	5.16
			0.20, 0.05	14.35	13.70	13.56	13.50
			0.20, 0.20	14.41	13.69	13.56	13.50
			0.60, 0.01	31.34	31.87	31.53	31.45
		.30,.10	0.01, 0.01	7.29	5.37	5.45	5.32
			0.20, 0.05	14.11	13.71	13.65	13.54
			0.20, 0.20	14.16	13.71	13.65	13.54
			0.60, 0.01	31.00	31.84	31.55	31.46
	High	.20,.20	0.01, 0.01	7.77	5.07	5.10	5.05
			0.20, 0.05	14.57	13.62	13.43	13.42
			0.20, 0.20	14.61	13.62	13.42	13.44
			0.60, 0.01	31.25	31.87	31.39	31.37
		.30,.10	0.01, 0.01	8.19	5.32	8.96	5.29
			0.20, 0.05	14.86	13.83	13.61	13.68
			0.20, 0.20	14.93	13.82	13.67	13.67
			0.60, 0.01	31.36	32.13	31.51	31.77
$J = 15$, unbalanced	Low	.20,.20	0.01, 0.01	6.59	4.41	4.55	4.45
			0.20, 0.05	10.49	8.95	8.88	8.86
			0.20, 0.20	10.54	8.95	8.87	8.87
			0.60, 0.01	19.87	19.46	19.15	19.13
		.30,.10	0.01, 0.01	6.54	4.77	5.03	4.82
			0.20, 0.05	10.47	9.27	9.19	9.11
			0.20, 0.20	10.50	9.27	9.20	9.13
			0.60, 0.01	19.84	19.87	19.40	19.23
	High	.20,.20	0.01, 0.01	6.92	4.36	4.48	4.39
			0.20, 0.05	10.86	8.95	8.84	8.89
			0.20, 0.20	10.93	8.95	8.84	8.89
			0.60, 0.01	20.01	19.55	19.17	19.15
		.30,.10	0.01, 0.01	7.24	4.59	4.95	4.60
			0.20, 0.05	11.25	9.08	9.18	9.03
			0.20, 0.20	11.31	9.08	9.17	9.04
			0.60, 0.01	20.39	19.66	19.45	19.34
